# Supply and Demand Drivers of Global Hydrogen Deployment in the Transition toward a Decarbonized Energy System

**DOI:** 10.1021/acs.est.3c03751

**Published:** 2023-11-07

**Authors:** Patrick O’Rourke, Bryan K. Mignone, Page Kyle, Bryan R. Chapman, Jay Fuhrman, Paul Wolfram, Haewon McJeon

**Affiliations:** † University of Maryland, College Park, Maryland 20742, United States; ‡ Pacific Northwest National Laboratory − Joint Global Change Research Institute, College Park, Maryland 20740, United States; § ExxonMobil Technology and Engineering Company, Annandale, New Jersey 08801, United States; ∥ KAIST Graduate School of Green Growth & Sustainability, Daejeon 34141, Korea

**Keywords:** low-carbon hydrogen, distributed hydrogen, hydrogen infrastructure, climate change mitigation, integrated assessment modeling

## Abstract

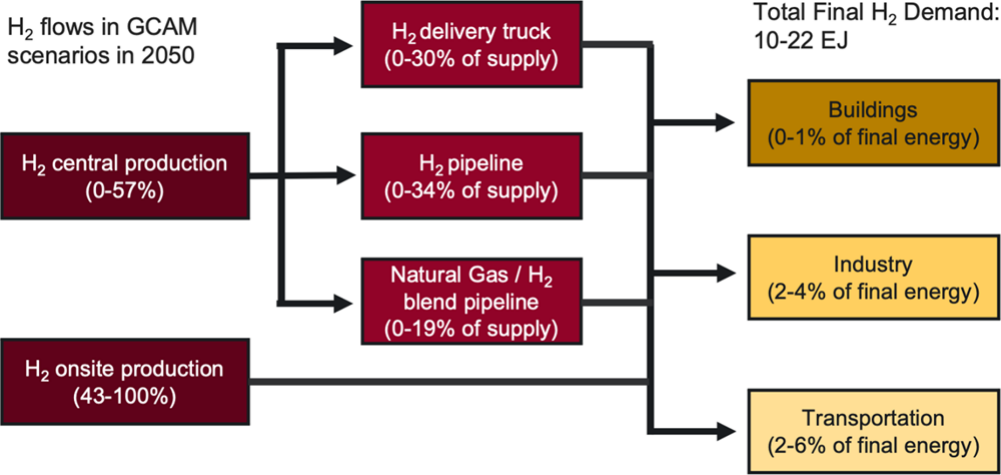

The role of hydrogen in energy system decarbonization is being actively examined by the research and policy communities. We evaluate the potential “hydrogen economy” in global climate change mitigation scenarios using the Global Change Analysis Model (GCAM). We consider major hydrogen production methods in conjunction with delivery options to understand how hydrogen infrastructure affects its deployment. We also consider a rich set of hydrogen end-use technologies and vary their costs to understand how demand technologies affect deployment. We find that the availability of hydrogen transmission and distribution infrastructure primarily affects the hydrogen production mix, particularly the share produced centrally versus on-site, whereas assumptions about end-use technology primarily affect the scale of hydrogen deployment. In effect, hydrogen can be a source of distributed energy, enabled by on-site renewable electrolysis and, to a lesser extent, by on-site production at industrial facilities using natural gas with carbon capture and storage (CCS). While the share of hydrogen in final energy is small relative to the share of other major energy carriers in our scenarios, hydrogen enables decarbonization in difficult-to-electrify end uses, such as industrial high-temperature heat. Hydrogen deployment, and in turn its contribution to greenhouse gas mitigation, increases as the climate objective is tightened.

## Abstract 2

The role of hydrogen in the energy transition is investigated. Integrated assessment modeling of a low-emissions future shows that hydrogen delivery infrastructure primarily affects the hydrogen production mix, whereas end-use technology costs primarily affect the scale of hydrogen deployment.

## 1 Introduction

The possibility of using hydrogen (H_2_) as an energy carrier has been discussed for nearly half a century. While literature in the 1970s and 1980s noted its potential to enhance national energy security,^1−4^ a more recent focus has been its role in decarbonization, particularly in end-use sectors that are difficult to electrify. Like electricity, hydrogen can be produced from numerous primary energy sources using different production processes. But to contribute to decarbonization goals, it must be produced using low-carbon technologies. Examples include natural gas reforming with carbon capture and sequestration (CCS) to make “blue” hydrogen^5^ or renewable-based water electrolysis (“green” hydrogen^6^) and biomass gasification.^7^ The last of these methods could be coupled with CCS to produce hydrogen with negative emissions.^8^

Once produced, low-carbon hydrogen could be used to reduce emissions in several areas of the global energy system. In the industrial sector, hydrogen could provide a low-carbon fuel for high-temperature process heat,^9^ be used directly as a reducing agent for iron (hydrogen direct reduced iron [DRI]),^10,11^ or provide low-carbon feedstocks for chemical and fertilizer manufacturing.^12^ Hydrogen could similarly mitigate carbon dioxide (CO_2_) emissions in the transportation sector–particularly in heavy-duty transportation (e.g., trucks^13^ and marine vessels^14^). Furthermore, blending low-carbon hydrogen with natural gas^15^ could lower the carbon intensity of technologies that consume natural gas. Given such versatility, hydrogen has captured the attention of policymakers, as evidenced by the publication of hydrogen “roadmaps” by over a dozen nations.^16−31^

Evaluating the role of hydrogen in the energy transition involves understanding where and when it could be competitive with other decarbonization options. The role of hydrogen has been examined in some energy system models in the context of net-zero scenarios but typically at the country or regional level and often using exogenous assumptions about end-use demand.^32−36^ For this reason, technology-rich integrated assessment models (IAMs) are particularly well suited to advance our understanding, given that they have global coverage and endogenously represent competition within end-use demand sectors as well as within energy supply sectors.^37,38^ Several IAM-based studies have considered the role of hydrogen in climate mitigation. Barreto et al. (2003) employed MESSAGE-MACRO and found that hydrogen could potentially provide nearly half of all global final energy by the end of the century.^39^ Other early IAM studies found a more modest role for hydrogen, typically centered on the transportation sector. Using MiniCAM, Edmonds et al. (2004) found hydrogen could supply a significant share of global transportation final energy under emissions mitigation but only if advanced technological assumptions were realized (e.g., low-cost production and higher efficiency fuel cells).^40^ van Ruijven et al. (2007) similarly found, using TIMER 2.0, that hydrogen could play a key role in the transportation sector but primarily in the second half of the century.^41^ Using TIAM-UCL, Anandarajah et al. (2013) also found that hydrogen could contribute meaningfully to the decarbonization of road-based transportation.^42^

Recent studies have begun to consider the role of hydrogen in more detail. Using the Global Change Analysis Model (GCAM), Lazarou et al. (2018) found that hydrogen deployment was higher under increasingly stringent climate constraints.^43^ McPherson et al. (2018), using MESSAGE, demonstrated how hydrogen could support variable renewable electricity (VRE) deployment by providing electricity storage and firm electricity generation capacity under mitigation.^44^ More recently, an assessment by Oshiro and Fujimori (2022) using the AIM/Technology model found that by midcentury hydrogen and hydrogen-derived energy carriers could provide up to ∼9% of global industrial final energy demand and up to ∼50% of global transportation energy needs in scenarios that limited warming to 1.5–2 °C.^45^ While hydrogen was not the main focus of their analysis, Luderer et al. (2022) also showed pathways in REMIND-MAgPIE with significant amounts of hydrogen deployment under 1.5–2 °C policy scenarios.^46^

However, to our knowledge, no IAM studies have explicitly evaluated the impact of hydrogen delivery infrastructure, even though hydrogen transmission and distribution (T&D) costs are expected to be substantial and therefore an important factor in determining the hydrogen production mix and its competitiveness with other energy carriers. And while electrolysis-based hydrogen has received attention from the IAM literature, it remains less clear whether this production pathway will utilize grid electricity or dedicated renewable energy, as well as whether supply will be centralized or distributed in general. In addition, many possible hydrogen end-use technologies discussed by policymakers and the research community have not been fully incorporated into models. Oshiro and Fujimori (2022) included hydrogen-derived synthetic fuel utilization for rail and ammonia-powered marine applications, but they did not model hydrogen fuel cell technologies for these end uses. Similarly, Lazarou et al. (2018) only included hydrogen use in aggregate industry and light-duty vehicles. Anandarajah et al. (2013) evaluated hydrogen usage for road-based transportation, but they did not evaluate nonroad modes of transport or other end-use sectors.

In this study, we represent all major low-carbon hydrogen production methods, including both distributed (on-site) and central production, multiple hydrogen delivery options, and a rich set of hydrogen end-use technologies within GCAM. Compared to prior studies, this work considers a wider range of hydrogen uses across all major end-use sectors and calls attention to the role of hydrogen T&D in driving choices about hydrogen supply chains. Our results suggest that outcomes such as the production mix, the share of on-site production, and hydrogen’s contribution to mitigation in different end uses vary with key assumptions.

## 2 Methods

This research utilized the Global Change Analysis Model (GCAM),^47^ a publicly available global hierarchical equilibrium IAM. Specifically, GCAM version 5.4^48^ was used as it was the most recent publicly available version of the model when this research began. Model enhancements were made to hydrogen-based technologies, as well as to other aspects of the energy system unrelated to hydrogen which are detailed in McJeon et al. (2021).^49^ GCAM links human and earth systems (the climate, water, land, and energy systems, as well as the economy), enabling examination of their interactions and responses to policy or technology changes. GCAM contains 32 energy and economic regions, 384 land areas, and 235 water basins, and the model projects various scenarios to the end of the century, running in five-year timesteps. Final energy demand in the model is driven by exogenous assumptions for income (GDP per capita) and population as well as endogenous changes in the cost of producing a given service (e.g., vehicle miles). The “middle-of-the-road” Shared Socioeconomic Pathway 2 (SSP2) assumptions^50^ are used in this study for population and GDP.

**Figure 1:**
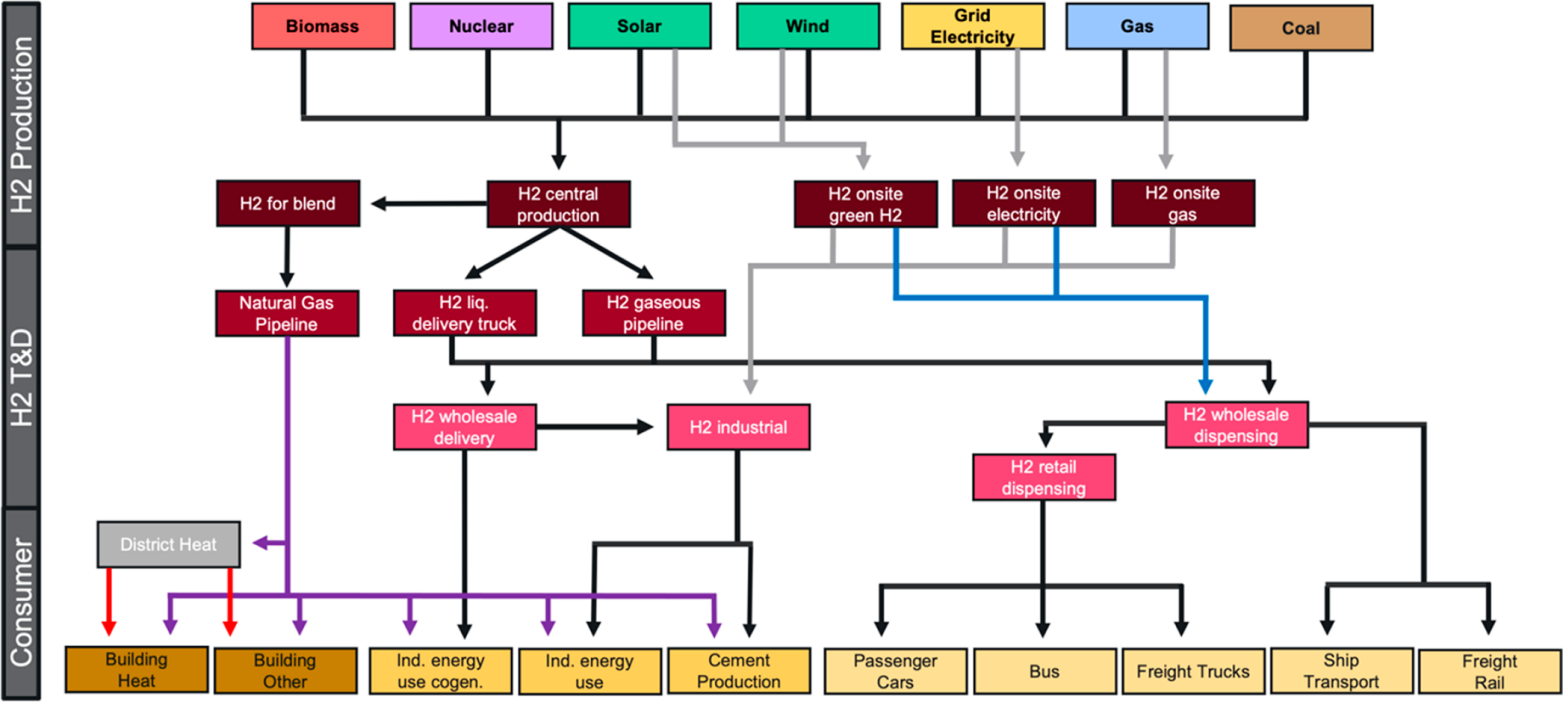
Representation of GCAMv5.4 hydrogen supply and demand, as modified for this study.

In each end-use sector, hydrogen competes with other energy carriers based on the levelized cost of supplying a given service (e.g., process heat for industrial needs) using a logit choice formulation.^51,52^ Hydrogen production technologies compete based on cost in a similar manner. When emissions are priced, as they are in climate mitigation scenarios, differences in emissions between technologies also affect competition. Emissions associated with resource production (e.g., natural gas) and energy conversion (e.g., electricity generation), along with emissions associated with energy requirements for T&D, are included within GCAM. In this way, GCAM accounts for the major sources of emissions across the life cycles of different fuels.

### 2.1 Hydrogen Production

Figure 1 shows the hydrogen structure in the updated version of GCAM used for this paper. This configuration allows hydrogen to be produced from biomass with or without CCS, fossil fuels with CCS, and multiple electrolysis pathways, including grid electrolysis, dedicated wind and solar electrolysis, and nuclear high temperature electrolysis (HTE). Hydrogen produced from fossil sources without CCS is not included given the focus on climate change mitigation scenarios in this study. Hydrogen can be produced centrally (i.e., at large-scale facilities that produce hydrogen that is subsequently delivered to end users) for all production methods. Alternatively, it can be produced on-site at the physical location of the end user, but on-site production is restricted to grid electrolysis, renewable electrolysis (green hydrogen), and natural gas with CCS (blue hydrogen). On-site blue hydrogen is assumed to be available only within industry given the scale required for CO_2_ capture. The representation of on-site green hydrogen assumes that renewable electricity is transmitted by the electricity grid from the VRE source to the end user that is producing hydrogen on-site. In contrast, central green hydrogen production is assumed to generate hydrogen at the VRE source, with hydrogen moved to the end user. While we assume both types of green hydrogen are produced synchronously with renewable electricity generation, the difference in their configurations allows one to investigate whether it may be advantageous to distribute green hydrogen rather than green electricity. On-site and central grid electrolysis is represented in a similar manner; however, these configurations are agnostic to the source of the electricity and therefore are assumed to operate at high capacity factors.

Hydrogen production assumptions are harmonized with the National Renewable Energy Laboratory’s (NREL’s) Hydrogen Analysis Production Models (H2A) version 3.2018,^53^ except for assumptions about electricity generation needed for green hydrogen, nuclear HTE, and bioenergy with CCS (BECCS), which are described below. Blue hydrogen is based on assumptions for gas steam methane reforming (SMR) with CCS. All forms of electrolysis utilize H2A’s data for proton exchange membrane (PEM) electrolyzers, which are considered compatible with intermittent electricity.^54,55^ Levelized costs are calculated using the regional capacity factors of their paired renewables. Solar panel and wind turbine nonenergy costs were aligned with GCAM’s default power sector assumptions, which are based on NREL’s Annual Technology Baseline (ATB) version 2019.^56^ Additional details regarding GCAM’s power sector methodology can be found in Muratori et al. (2017).^57^ Nuclear HTE assumptions are also informed by NREL’s H2A models, but because there is no 2018 H2A version for this technology, we utilized the 2008 H2A version,^58^ the most recent publicly available, and updated the reactor costs using ATB version 2019. The H2A models provide assumptions for biomass hydrogen without CCS, so the additional costs and energy requirements for CCS were added to define the BECCS hydrogen technology. For all hydrogen production technologies coupled with CCS, capture fractions increase from 91% in 2020 to 93% in 2050.

Table S1 contains the levelized nonenergy costs for hydrogen production as well as feedstock and/or energy requirements for each production technology. Given the global scope and projection horizon, specific policy measures related to hydrogen that have been adopted by some countries, such as the Inflation Reduction Act in the US, are not included in this analysis. Regional policy measures, combined with differences in market conditions across regions, may lead to differences in the realized costs of hydrogen production technologies. Additional hydrogen production assumptions are discussed in the Supporting Information (S.1.A).

### 2.2 Hydrogen Transmission and Distribution

As a means of delivering centrally produced hydrogen, we represent liquid hydrogen truck and gaseous hydrogen pipeline T&D (both are referred to as dedicated hydrogen T&D in this study). The cost and efficiency assumptions for hydrogen delivery, found in Table S2, are based on Argonne National Laboratory’s Hydrogen Delivery Scenario Analysis Model (HDSAM) version 3.1.^59^ Hydrogen delivery from central production facilities is assumed to require 100 km of transmission to the end user, except for central green hydrogen production, which is assumed to require 500 km of transmission. The additional distance reflects an assumption that electrolyzers would be colocated with large wind or solar farms, which are generally sited where the renewable resource quality is highest, not near hydrogen demand centers. The first year of hydrogen pipeline operation in cases that allow pipeline delivery is 2030, reflecting the fact that it would take time to build out the hydrogen pipeline network. Trade of hydrogen between GCAM’s 32 native regions via continental pipelines or oceanic-based tankers is not included within this analysis.

**Figure 2:**
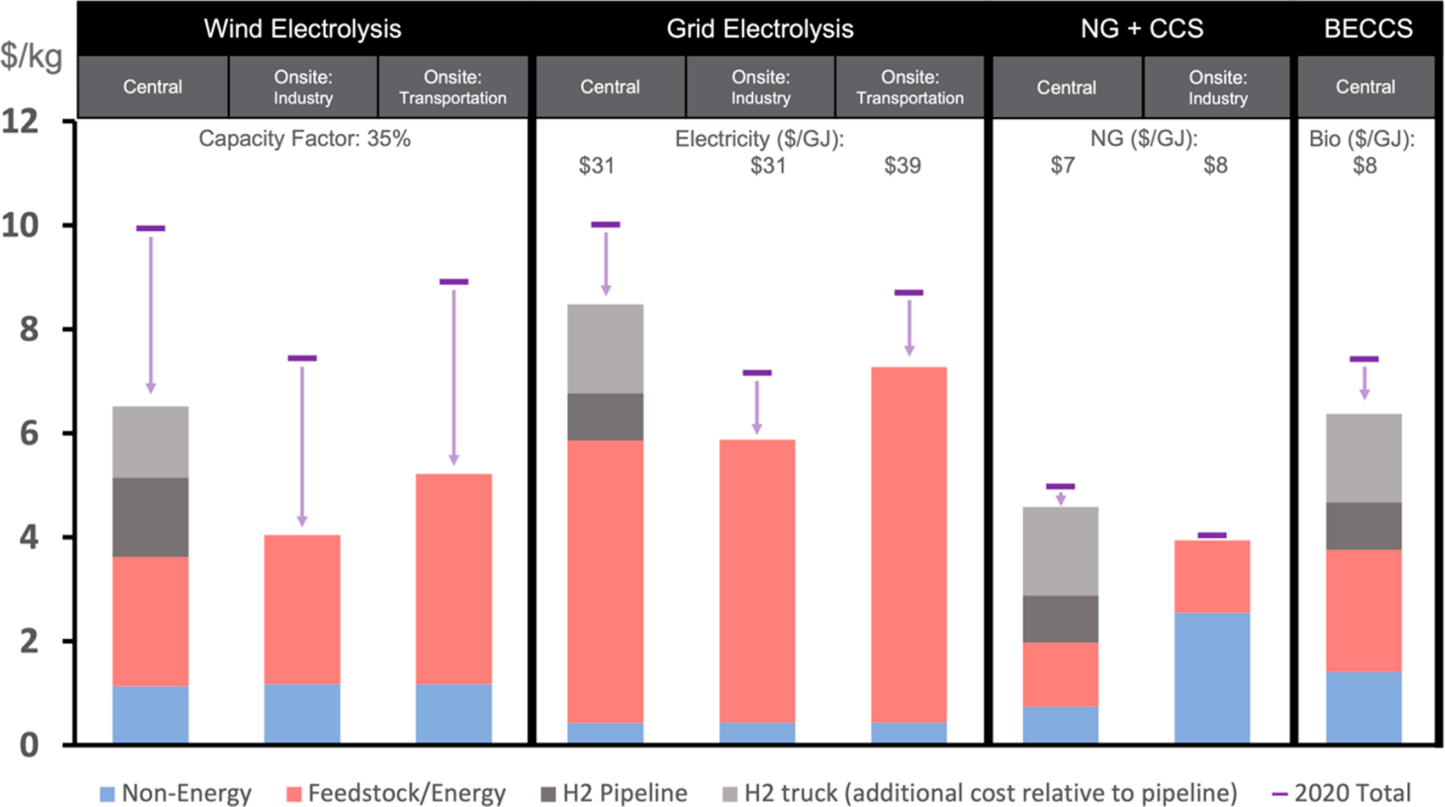
Levelized Cost of Hydrogen (LCOH) in 2020 dollars per kg of H_2_ in 2050. The LCOH for a given production method varies regionally and across scenarios due to differences in the projected prices of delivered energy as well as renewable capacity factors. LCOH values presented here use illustrative delivered energy prices and renewable capacity factors, which are within the model’s ranges for these assumptions and projections across regions. “Non-Energy” costs refer to levelized capital and operating expenses. “Feedstock/Energy” costs refer to the costs associated with the feedstock and other energy inputs. The “Feedstock/Energy” cost for electrolysis is primarily the cost of electricity, which is either purchased (e.g., grid electrolysis) or produced on-site (e.g., central wind electrolysis). The total cost of delivering hydrogen via trucks is equal to the sum of costs for the “H_2_ pipeline” and “H_2_ truck (additional cost relative to H_2_ pipeline)”. The “2020 Total” is the total levelized cost for a given production technology in 2020.

The modified version of GCAM also allows blending of hydrogen into natural gas T&D networks as a third method of transporting hydrogen to consumers. The share of hydrogen blended into natural gas pipelines could be limited by multiple factors, including the potential for hydrogen to degrade the structural integrity of pipelines and compatibility with end-use technologies. This blend tolerance varies not only by pipeline type (materials) but also by end-use technology. A wide range of blend limits ranging from 2 to 30% has been reported in the literature.^9,60−62^ In the High Demand cases (discussed below), we assume a 20% blend by volume, or roughly a 7% blend by energy content,^63^ phased into natural gas distribution networks such that all new gas technologies in the relevant end-use sectors are consuming the blend by midcentury. In the Reference Demand cases (discussed below), we do not allow for any hydrogen blending with natural gas. See section S.1.B. for additional details on hydrogen T&D and blend assumptions. Figure 2 provides representative 2050 levelized costs of hydrogen for several key technologies in GCAM.

### 2.3 Hydrogen End Uses

End-use consumption of hydrogen is limited to final energy applications in GCAM: cement production process heat, other industrial energy uses (e.g., process heat and cogeneration of power plus heat), passenger cars (multiple size classes), buses, freight trucks (multiple size classes), marine vessels (domestic and international), and freight rail transport. We use the term ‘other industrial energy use’ to refer to GCAM’s “industrial energy use” sector, which considers all energy use for industrial applications that are not already explicitly represented as separate sectors within this version of the model (note that energy used for mining, construction, and agriculture is included in ‘other industrial energy use’). While hydrogen-powered aviation has also been considered as a potential future hydrogen application, it was not included in this analysis, as there is a high level of uncertainty around this technology, making it difficult to identify plausible techno-economic assumptions. Given this study’s focus on hydrogen’s use for final energy, we do not consider hydrogen utilization for feedstocks (e.g., chemicals or hydrogen-derived fuels) or within the electricity sector. Hydrogen storage costs and energy requirements are included for refueling stations in the transportation sector (based on HDSAM) but not for the industrial sector given that comparable costs (e.g., on-site fuel storage) for other industrial technologies are not included within GCAM. When available, the hydrogen/natural gas blend can be consumed by specific end uses: cement manufacturing (process heat), other industrial energy uses (process heat and cogeneration), and building and industrial heat (district heat, direct residential and commercial heat, as well as other building energy needs such as water heating and cooking). Given that the blend limit and decision to phase in the blend are based on delivery tolerance and end-use technology thresholds, we do not include additional costs for end users to consume the blend above the cost differential of the fuel. However, we include the additional cost of compressing hydrogen for delivery through natural gas pipelines.

### 2.4 Energy Transition Scenarios

Table 1 describes the core scenarios examined using GCAM, which are defined by supply and demand assumptions. In this study, GCAM is coupled with the MAGICCv5.3 climate model.^64^ All core scenarios limit warming below 2 °C through the century, with a terminal temperature of 1.75 °C, by imposing a uniform global carbon price beginning in 2025 which rises 3% annually. Non-CO_2_ emissions (converted to CO_2_e emissions using the GWP-100) are abated using a marginal abatement cost curve to estimate the amount of abatement that would occur at the realized carbon price each year.^65^ The starting values of the carbon price are adjusted to attain the climate objective. We also consider a reduced set of scenarios that limit warming to 1.5 °C (with a terminal temperature of 1.35 °C; see section S.3). Temperature outcomes were also calculated using MAGICC v7.5.3 under the “IPCC AR6 WGI (Probabilistic)” setting, returning a median 1.84 °C temperature in 2100 for the below 2 °C scenarios and a 1.5 °C median for the more stringent climate objective.^66^ For the below 2 °C climate objective, we consider three options for hydrogen delivery:

(1) No dedicated hydrogen T&D infrastructure available (only on-site production allowed)
(2) Hydrogen delivery by hydrogen trucks is allowed (in addition to on-site production)
(3) Hydrogen delivery by dedicated pipelines is allowed beginning in 2030 (in addition to truck delivery and on-site production).

**Table 1:**
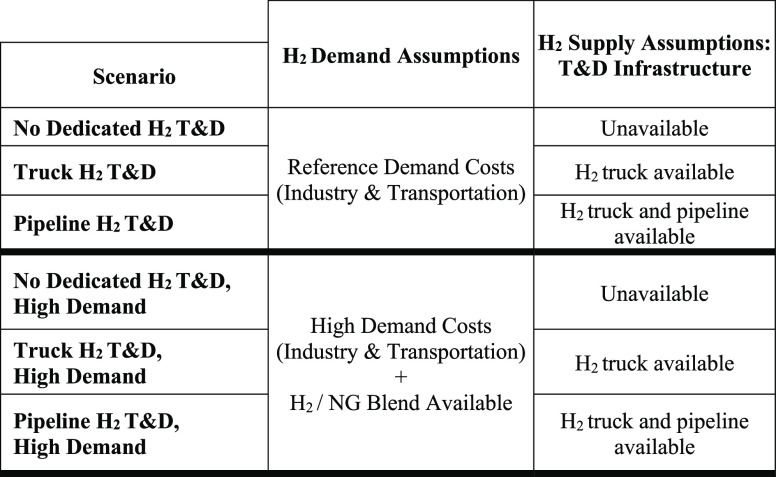
Core Scenarios Examined in This Study^a^

a T&D refers to transmission and distribution. All core scenarios limit warming below 2 °C through the century. Additional sensitivities, including scenarios that limit warming to 1.5 °C, can be found in the Supporting Information (see sections S3–S4).

We also consider two sets of assumptions for hydrogen demand technologies:

(1) *Reference Demand*: capital costs for hydrogen technologies in the transportation and industrial sectors remain constant after their first year of availability (see Table S3 for availability years).
(2) *High Demand*: capital costs for hydrogen technologies in the transportation sector decline after their first year of availability; nonenergy costs for hydrogen technologies in the industrial sector also decline, attaining a 25% cost reduction between 2020 and 2050; and hydrogen blending into natural gas networks is allowed.

In addition to 1.5 °C sensitivity cases, we consider several other sensitivities in the Supporting Information. Two scenarios limit bioenergy consumption globally, with one further constraining the amount of globally available carbon sequestration (“Bio Low” and “Bio Low/CCS Low”, S.4.A). We also include sensitivity scenarios (S.4.B–S.4.C) that restrict hydrogen production to central facilities and include direct air capture (DAC).

## 3 Results

### 3.1 Global Hydrogen Production through Midcentury

**Figure 3:**
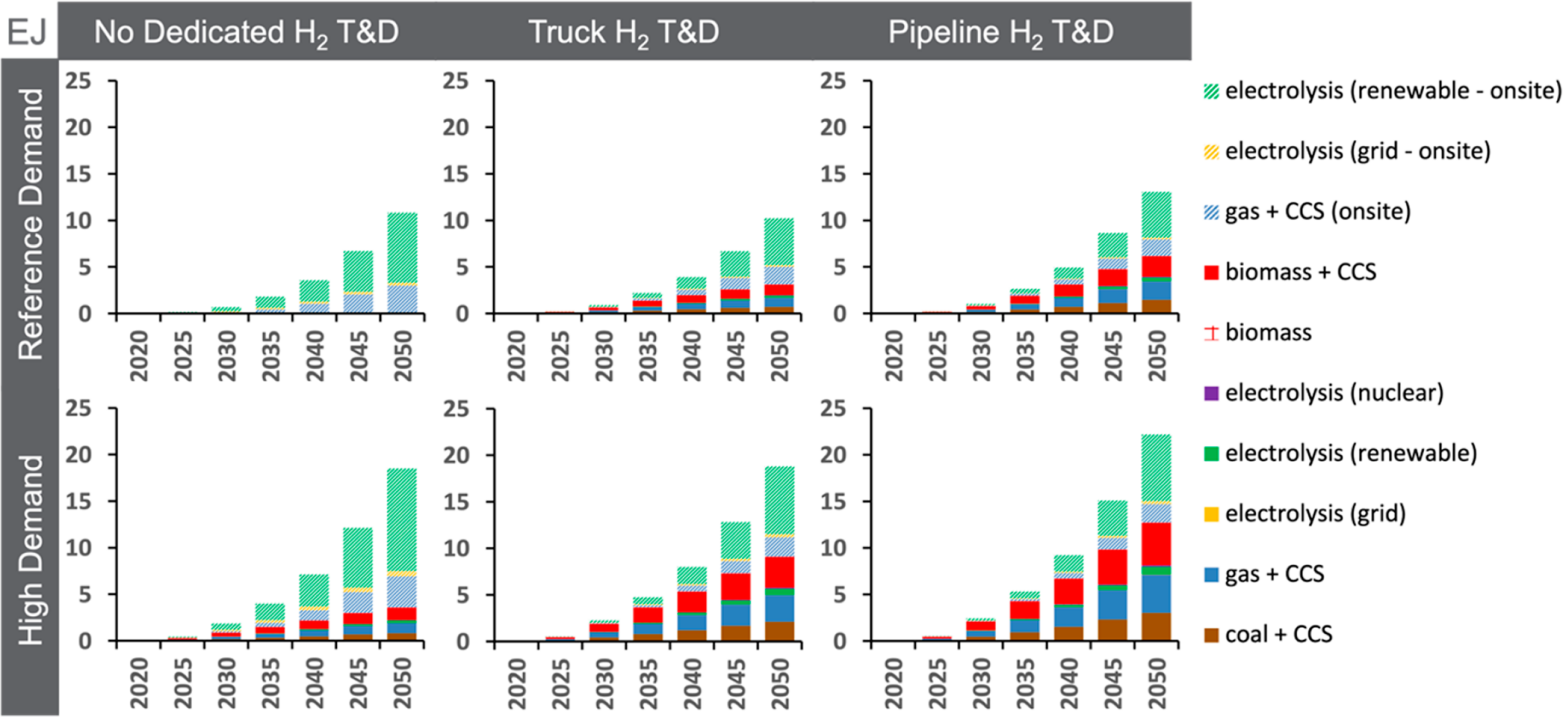
Annual global hydrogen production (EJ) by technology through midcentury for the core scenarios. Centrally produced hydrogen is shown using solid colors, whereas on-site production is shown using diagonal hashed colors. For the hydrogen production mix by end-use sector, see Figure S4. The production mix also varies by region, primarily due to differences in regional energy/feedstock costs and renewable capacity factors.

In all core scenarios, global hydrogen production remains below 2.5 EJ through 2030, but it reaches 10–22 EJ across scenarios by 2050 (Figure 3). For a sense of scale, in 2050, this equates to roughly 9–20% of total final energy consumed within just the transportation sector in 2015 (113 EJ). In each case, green hydrogen provides the largest share of production by midcentury–ranging from 36% to 69% of all hydrogen produced. The vast majority (>90%) of green hydrogen is produced on-site due to its lower cost (Figure 2). On-site production has slightly higher nonenergy costs (since electrolyzer costs are lower for central production due to economies of scale) as well as higher feedstock/energy costs (primarily from the cost of delivering solar- or wind-based electricity produced offsite), but these additional costs are more than offset by the cost savings from avoiding hydrogen T&D. On-site green hydrogen production (and its share of total production) further increases when warming is limited to 1.5 °C (section S.3).

A key insight from this analysis is that on-site production of hydrogen, which is dominated by on-site green hydrogen in all GCAM scenarios, is a substantial proportion of total hydrogen production. In 2050, on-site production of hydrogen accounts for more than half of total hydrogen production in all but one of the six scenarios (in which it accounts for 43%). Still, the role for on-site production is not limited to green hydrogen, as industrial on-site blue hydrogen is also competitive with other technologies (Figure 2). Blue hydrogen accounts for 24–29% of total hydrogen production in 2050. But even when central blue hydrogen is available (with dedicated hydrogen T&D), 33–66% of all blue hydrogen is produced on-site.

Interestingly, the *scale* of hydrogen deployment is largely unaffected by the availability of dedicated hydrogen T&D infrastructure. Under Reference Demand assumptions, allowing both pipelines and truck delivery increases total hydrogen production by <2 EJ in 2050. While not materially impacting the scale of hydrogen production, dedicated hydrogen T&D enables delivered hydrogen to play a larger role. For instance, central production accounts for 0–19% without dedicated T&D (blending with natural gas allows for some central production), 30–48% when truck delivery is available, and 47–57% when pipelines are also available. High Demand scenarios exhibit higher overall hydrogen deployment than their corresponding Reference Demand scenarios but a similar production mix. As shown in Figure S11, the scale of hydrogen deployment is also found to be strongly sensitive to the climate objective, more than doubling by midcentury under scenarios limiting warming to 1.5 °C (30–48 EJ).

The introduction of dedicated hydrogen T&D opens the door for hydrogen produced centrally using natural gas with CCS, coal with CCS, biomass (including BECCS), and nuclear to contribute to emissions mitigation. Among these options, natural gas with CCS and BECCS are the most competitive (Figure 2). Although BECCS has a higher total levelized cost than industrial on-site green and blue hydrogen when there is no carbon price, BECCS provides a source of negative emissions and receives a subsidy under carbon policies. By 2050, BECCS hydrogen ranges from 1.2 to 4.6 EJ or 11 to 21% of total hydrogen production (with dedicated hydrogen T&D). However, BECCS hydrogen is only a small share of total biomass consumption (4% of modern biomass consumption in 2050 when pipelines are available under the Reference Demand assumptions), because other BECCS options in GCAM (e.g., in power, liquids production) are more attractive, either due to cost or their potential deployment scale. We consider two sensitivity scenarios–one in which biomass is limited and a second in which carbon sequestration is also limited–in the Supporting Information (section S.4.A). Lastly, the production mix (shares of each production type) as well as hydrogen production volumes in our core scenarios are well within the ranges found in scenarios limiting warming to 2 °C in the IPCC AR6 database^67^ (Figures S5 and S6).

### 3.2 Global Hydrogen Consumption through Midcentury

**Figure 4:**
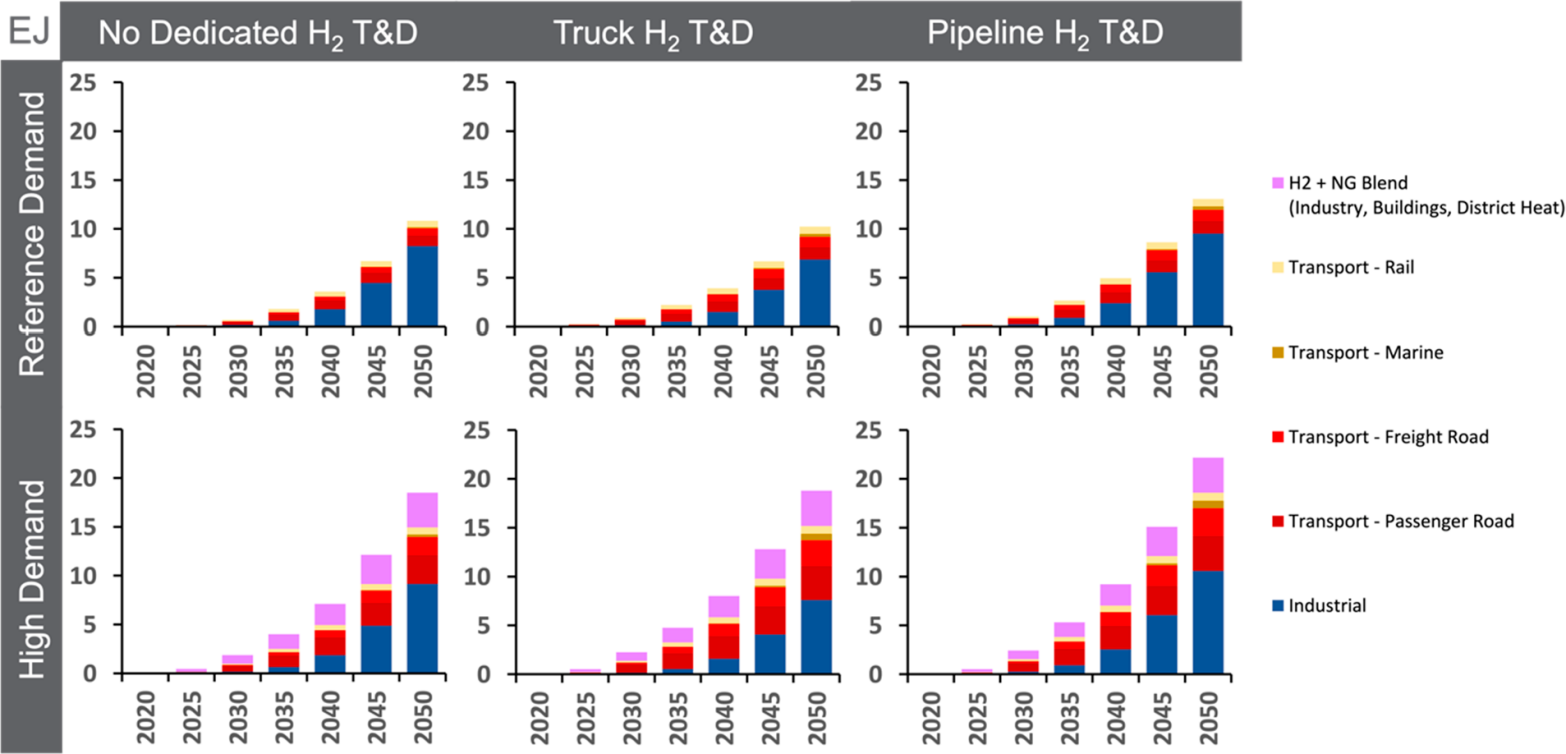
Annual global hydrogen consumption (EJ) by end-use sector through midcentury for the core scenarios. Only the amount of hydrogen within the hydrogen/natural gas blend is included in the pink part of each bar.

Under both Reference and High Demand scenarios, industrial hydrogen demand by 2050 is comparable to or larger than transportation hydrogen demand, accounting for 40% to 76% of hydrogen consumption or ∼7 to 11 EJ (or up to 12.5 EJ when including hydrogen blended with gas, Figure 4 and Table 2). Total hydrogen consumption in the High Demand scenarios is higher than in the corresponding Reference Demand scenarios by 8–9 EJ (or 70–84%) in 2050. Assumed cost reductions in hydrogen-based transportation lead to a noticeable increase in the level of hydrogen consumption for road transportation. However, even with High Demand assumptions and the ability to deliver hydrogen in pipelines, only 7% of freight road and 5% of international shipping service demand (tonne-km) are fulfilled by hydrogen technologies in 2050. Most of the remaining demand (66% of freight road and 94% of international shipping) is met by technologies that consume liquid fuels.

**Table 2:** Annual Global Hydrogen Consumption (EJ) by Each End-Use Sector in 2050 for Two of the Core Scenarios^a^

	**Scenario**							
	**No Dedicated H_2_ T&D, Reference Demand**				**Pipeline H_2_ T&D, High Demand**			
**Sector**	Final energy (EJ)	H_2_ demand (EJ)	% final energy	% service output	Final energy (EJ)	H_2_ demand (EJ)	% final energy	% service output
**Industry**	**278.9**	**8.2**	**3.0%**	**—**	**281.4**	**12.5**	**4.4%**	**—**
Cement	15.3	1.2	7.6%	—	15.3	1.4	9.1%	—
Other industrial energy use	196.3	7.1	3.6%	—	198.5	11.1	5.6%	—
**Transportation**	**135.0**	**2.6**	**1.9%**	**—**	**134.2**	**8.0**	**6%**	**—**
Passenger Vehicle	39.0	0.6	1.4%	1.5%	38.6	3.0	7.7%	8.3%
Bus	6.0	0.6	9.8%	22.1%	6.0	0.7	11.1%	24.6%
Freight Road	47.8	0.7	1.5%	1.7%	47.1	2.8	6.0%	6.9%
Freight Rail	3.8	0.7	17.3%	16.5%	4.0	0.8	20.7%	19.7%
Domestic Shipping	2.3	0.03	1.4%	1.5%	2.3	0.2	8.2%	8.7%
International Shipping	13.2	0.1	0.6%	0.7%	13.2	0.6	4.4%	5.5%
**Buildings**	**171.0**	**0**	**0%**	**—**	**171.9**	**1.5**	**0.9%**	**—**
Heat	33.2	0	0%	—	33.5	0.9	2.7%	—
Other	121.3	0	0%	—	121.7	0.6	0.5%	—
**Total**	**584.9**	**10.8**	**1.9%**	**—**	**587.4**	**22.0**	**3.7%**	**—**

a Values (EJ and %) are rounded to the nearest tenth if larger than or equal to 0.05 (otherwise values are presented to the nearest hundredth). ‘Other industrial energy use’ refers to the GCAM sector “industrial energy use”. The subsector “Other” within the buildings sector refers to the sum of GCAM’s “residential other” and “commercial other” subsectors. Hydrogen contained within the hydrogen/natural gas blend is included within these values. Subsectors will not sum to the sector total given that there are subsectors that do not consume hydrogen and are thus not reported here (e.g., building cooling, aviation, industrial feedstocks, N fertilizer production, and energy for water).

Compared to other energy carriers, hydrogen provides a smaller share of total final energy at midcentury (2–4%), with a similar share in transportation (2–6%) and industry (2–4%). The shares of hydrogen in final energy in the transportation and industrial sectors in our scenarios are well within the ranges for these outcomes assessed by the IPCC AR6^55^ with comparable climate objectives (Figure S7). Despite being a small portion of total final energy in 2050, hydrogen deployment could further increase after midcentury or before midcentury under more stringent climate objectives. For example, while the role of hydrogen in international shipping is modest through 2050 in the core scenarios, we find hydrogen-fueled international ships provide 65–89% of this subsector’s final energy by 2100 (SI section S.2.A). In addition, hydrogen provides 58–90% of the final energy in international shipping by 2050 in scenarios that limit warming to 1.5 °C (Table S7).

Within industry, most hydrogen is expected to be used in processes that require high-temperature heat (>400 °C). IEA (2017) noted that high-temperature heat accounted for 48% of global industrial heat requirements or nearly 36% of industrial energy demand (not including feedstocks).^68^ These applications are contained within GCAM’s cement and “industrial energy use” sectors, in which hydrogen deployment is relatively modest through midcentury across scenarios (e.g., <10% of final energy for cement). However, these subsectors decarbonize more substantially after midcentury in the core scenarios or before midcentury when the climate objective is tightened.

In the High Demand scenarios, hydrogen blended into the natural gas pipeline network accounts for 36–47% of hydrogen consumption (∼0.9 EJ) in 2030. But by midcentury, the proportion of hydrogen consumption from the blend shrinks to 16–19%. This is because the approximate 7% emissions reduction from a 20% hydrogen blend by volume^51^ is not sufficient to avoid the increasing penalty on carbon emissions from natural gas. And although the blend (hydrogen + natural gas) represents ∼38% of final energy for heating buildings across the High Demand scenarios in 2050, the hydrogen contained within the blend represents < 3% of final energy for building heating and a smaller share of total buildings final energy. See Table 2 for additional details about hydrogen consumption and end use.

### 3.3 Connecting Hydrogen Supply and Demand: Delivery Pathways to End Uses

**Figure 5:**
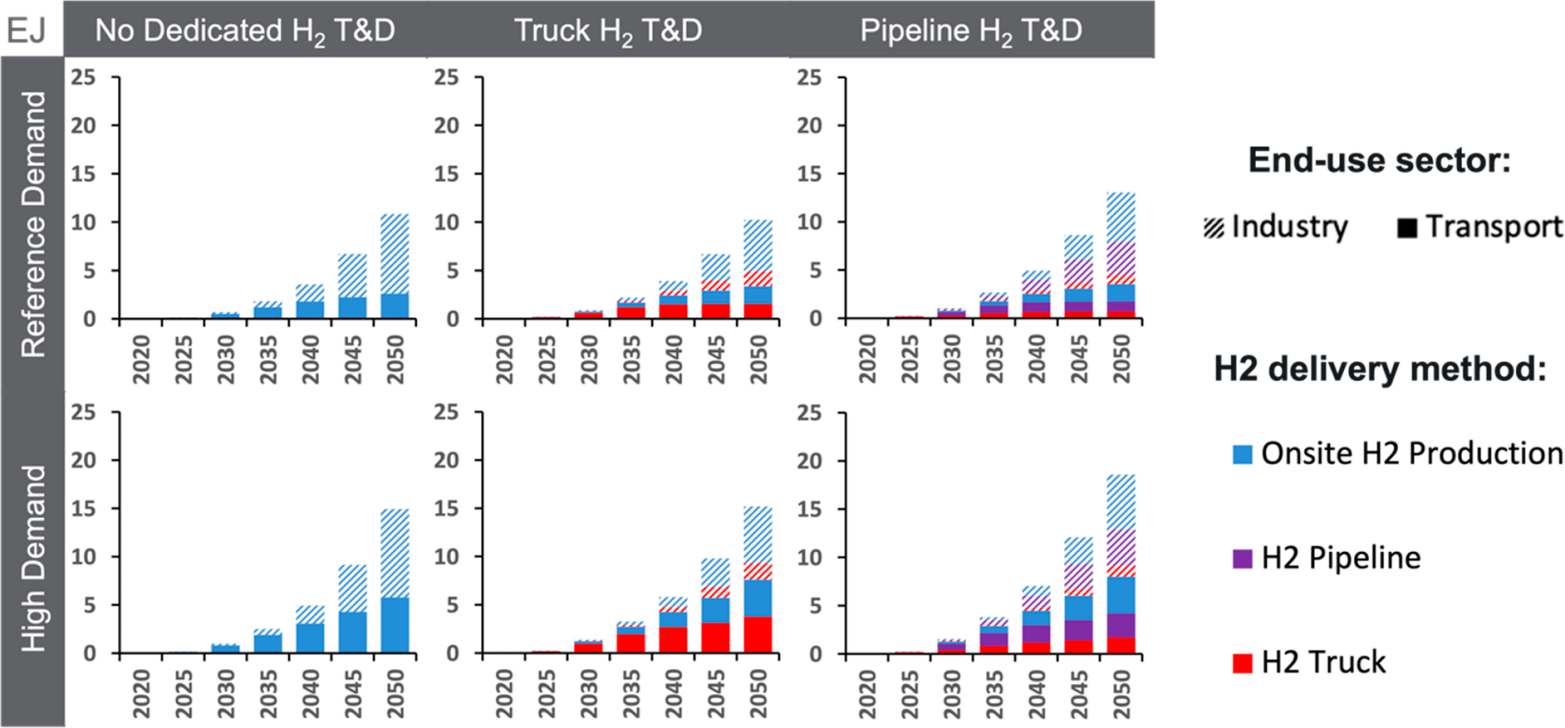
Annual global hydrogen consumption by delivery method to end-use sectors through midcentury for the core scenarios. The figure does not include hydrogen blended with natural gas and delivered by the natural gas pipeline network.

Figure 5 shows hydrogen consumption by each major end-use sector and delivery pathway. Truck and pipeline transport can be significant, with pipeline transport generally favored over truck transport when both are available due to differences in assumed costs. However, in all cases, on-site hydrogen production is larger than hydrogen delivered by truck or pipeline (as well as the amount blended into the natural gas networks). Furthermore, consumption by industry is comparable to and often larger than consumption by the transportation sector.

These results are relevant to the evaluation of hydrogen leakage potential, which is of increasing interest, in part, due to the indirect impact of hydrogen emissions on the climate.^69,70^ Other work has begun to evaluate which areas of the supply chain would have greater or lesser leakage risk, with production, transportation by pipeline, and industrial uses appearing to have lower risk than truck transport or use in transportation.^71^

A wide range of hydrogen leakage rates across hydrogen supply and demand has been published in the literature.^71−74^ The hydrogen leakage rate could be reduced if hydrogen were produced on-site, with estimates suggesting less than half a percent lost from electrolysis and SMR^71^ (not withstanding losses from storage). Leakage potential is also thought to be lower for dedicated gaseous pipeline delivery than for liquid hydrogen truck T&D.^71,72^ Our results show that when pipelines are available, truck delivery plays a smaller role. When both hydrogen T&D options are available, 13–15% of total hydrogen is distributed by trucks and 34% by pipelines in 2050 (not including blended hydrogen). Thus, leakage potential is likely reduced with pipeline availability relative to a scenario in which T&D is limited to trucks. Greater leakage risk is likely to occur when hydrogen is delivered by truck to the transportation sector, because, after hydrogen is loaded onto trucks, additional hydrogen can be lost at refueling stations.^71^ Yet, we find truck delivery to the transportation sector to be a small portion of total hydrogen consumption across scenarios (less than 9% in 2050, not including blended hydrogen). Further study of leakage rates across different production, delivery, and consumption pathways would help to inform an assessment of overall H_2_ leakage potential and options to mitigate leakage.

## 4 Discussion

This study investigated hydrogen’s potential contribution to decarbonization focusing on the role of hydrogen T&D infrastructure availability, end-use technology costs, and climate policy stringency in GCAM scenarios. Results from these scenarios provide several insights about the potential role for hydrogen in an energy transition. On the supply side, when distributed production options are represented, the availability of hydrogen T&D primarily affects the production mix rather than the scale of hydrogen deployment. In effect, on-site production places a floor on production in our scenarios and is found to be cost-effective (accounting for over half of production in five of the six core scenarios) even when T&D infrastructure is available to deliver centrally produced hydrogen. Consistent with other recent IAM analyses that have noted the potential role for electrolysis-based hydrogen,^45^ we find that the largest share of on-site production is from green hydrogen due to its modularity. However, the role of on-site blue hydrogen in industry is also significant.

The large portion of on-site generation coming from green hydrogen is notable as modular electrolyzers are “granular” technologies (smaller in physical and/or economic scale per unit), and it has been demonstrated that increased granularity can be associated with quicker learning and diffusion timeframes as well as lower investor risk.^75^ On-site production of low-carbon hydrogen (whether blue or green) could also avoid aspects of the ‘three-sided chicken-and-egg’ problem,^76^ that is the need to simultaneously foster demand, supply, and delivery infrastructure. This is because the supply and demand for hydrogen would be balanced within a facility (i.e., a consumer need not wait for a centralized supply of hydrogen, which may not materialize until demand is apparent). While hydrogen exports may expand the role of centrally produced hydrogen, the inclusion of hydrogen trade across GCAM regions is not likely to significantly change our results since the high cost of moving hydrogen regionally already limits the deployment of the centralized supply.

On the demand side, industrial use of hydrogen is found to be greater than the sum of all transportation uses in most of our core scenarios, reflecting in part the overall size of final energy demand in these sectors (industrial final energy demand is about twice transportation final energy demand in 2050). However, in scenarios with lower end-use costs, the hydrogen share of transportation final energy is greater than the hydrogen share of industrial final energy, suggesting that hydrogen demand is sensitive to end-use technology costs. Indeed, we find that the overall amount of deployment is driven more by end-use technology costs than by assumptions about T&D infrastructure. In addition, we find that overall hydrogen deployment is sensitive to increased climate policy stringency (increasing both in absolute terms and as a share of final energy), suggesting that hydrogen will remain a key focus in policy and planning related to the energy transition.

We also note that the small share of hydrogen transported by truck and delivered to the transportation sector across our scenarios may diminish the risk of leakage relative to scenarios in which those pathways are more dominant. Leakage risk would also presumably be lower when a larger share of hydrogen is produced and consumed on-site. Given the pathways by which hydrogen is produced and consumed in GCAM scenarios and the increasing discussion of hydrogen leakage, it would be helpful for future research to consider leakage at each stage of the supply chain explicitly, such that leakage assumptions are consistent with the pathways. Finally, while our focus has been on hydrogen demand by energy end uses, questions remain about the role of hydrogen in the power sector and in the production of hydrogen-derived fuels, which will be a focus of future GCAM development.

## Data Availability

The GCAM model and the data system are available as an open-source package (see https://github.com/JGCRI/gcam-core). The customized version of the model used in this study, as well as the scenario data, can be accessed at 10.5281/zenodo.8329505.

## Supporting Information Available

Additional details on methodology for hydrogen production, delivery, and end-use technologies, as well as supplementary results for core scenarios and supporting sensitivities (PDF)
