# Supplementary material for: Supply and Demand Drivers of Global Hydrogen Deployment in the Transition toward a Decarbonized Energy System
Source: Environ Sci Technol. 2023 Nov 7;57(48):19508–18. doi: 10.1021/acs.est.3c03751 (PMC10702436; doi:10.1021/acs.est.3c03751)
Supplement: Supplementary file 1 — es3c03751_si_001.pdf [file es3c03751_si_001.pdf]

**Supply and demand drivers of global hydrogen deployment in the transition towards a decarbonized energy system**

***Supplementary Information***

Patrick O'Rourke[1,2], Bryan K. Mignone[3], Page Kyle[2], Bryan R. Chapman[3],
Jay Fuhrman[2], Paul Wolfram[2], Haewon McJeon[2, 4, *]

[1] University of Maryland, College Park, MD, 20742
[2] Pacific Northwest National Laboratory – Joint Global Change Research Institute, College Park, MD, 20740, USA
[3] ExxonMobil Technology and Engineering Company, Annandale, New Jersey, 08801, USA
[4] KAIST Graduate School of Green Growth & Sustainability, Daejeon, Korea

* Correspondence to Haewon McJeon (hmcjeon@kaist.ac.kr)

Summary of the supporting information: 35 pages, 18 figures, and 8 tables

**Table of Contents**

**S.1. Methodology**……………………………………………………………………… S3

***S.1.A. Hydrogen production***…………………………………………………….. S3

***S.1.B. Hydrogen Transmission & Distribution***………………………………… S7

***S.1.C. End-use sector model modifications***……………………………………... S11

**S.2. Additional results for the core scenarios**………………………………………... S13

***S.2.A. Hydrogen in the second half of the century – below 2°C***…………........... S21

**S.3. Sensitivity: limiting warming to 1.5°C**……………………………………...... S23

**S.4. Additional sensitivities**……………………………………………...………. S26

***S.4.A. Sensitivity: biomass and CCS limited (2°C)***……………………………… S26

***S.4.B. Sensitivity: no onsite $H_2$ production (2°C)***…………………………...….. S29

***S.4.C. Sensitivity: direct air capture (DAC) available (2°C)***…………………… S31

## S.1. Methodology

### *S.1.A. Hydrogen production*

We allow hydrogen to be produced from the following low-carbon technologies: biomass with or without CCS, fossil fuels with CCS, as well as from multiple electrolysis pathways, including grid electrolysis, dedicated wind and solar electrolysis, and nuclear high temperature electrolysis (HTE). Unabated fossil pathways are not included. Hydrogen can be produced centrally (i.e., at a large-scale facility that subsequently distributes hydrogen to end users) for all methods of production. Alternatively, it can also be produced onsite at the physical location of the end user, but then the production methods are restricted to grid electrolysis, renewable electrolysis (green hydrogen), and natural gas steam methane reforming (SMR) with CCS (blue hydrogen).

Hydrogen production levelized non-energy costs (CAPEX and non-fuel OPEX) and energy requirement assumptions are harmonized with the U.S. Department of Energy's (DOE) National Renewable Energy Laboratory's (NREL) Hydrogen Analysis Production Models (H2A) version 3.2018[1], except for the renewable electricity generation for green hydrogen, nuclear high temperature electrolysis (HTE), and biomass with CCS (discussed in more detail below). H2A's 'current' assumptions were applied to 2015 for model inputs. The H2A models also provide 'future' assumptions for 2040. Improvement rates for levelized non-energy cost and energy requirements were constructed from the two years of H2A data to provide interim year assumptions (2020-2035). After 2040 these improvement rates are continued until a maximum of 10% additional improvement is reached (afterwards costs and efficiency are held constant). See *Table S1* for assumed hydrogen production cost and energy requirements.

Biomass with CCS and future year assumptions for coal with CCS are not provided by H2A version 3.2018. Biomass with CCS assumptions for 2015 were therefore derived by adding the additional costs and energy requirements of CCS. This was done by calculating the ratio between GCAM's power sector's integrated gasification combined cycle (IGCC) with and without CCS biomass technologies and applying these to H2A's biomass without CCS technology. The increased costs and decreased efficiency for biomass with CCS, compared to biomass without CCS, were reduced over time (in line with the difference between costs and efficiencies for GCAM's power sector biomass IGCC technologies. Additional details regarding GCAM's power sector methodology can be found in Muratori et al (2017).[2]

Both grid and green hydrogen (for central and onsite production) utilize H2A's data for proton exchange membrane (PEM) electrolyzers, which are considered appropriate for intermittent electricity.[3] While the electrolyzer costs for green hydrogen are derived from the H2A assumptions, the levelized costs are calculated using the regional capacity factors of their paired renewables. This was done by determining the relationship between capacity factor and electrolyzer non-energy costs using H2A data (for central and distributed electrolysis). These relationships are depicted in *Figures S1 and S2.* Solar panel and wind turbine levelized non-energy costs for green hydrogen production were aligned with GCAM's default power sector assumptions, which are based on NREL's Annual Technology Baseline (ATB) version 2019.[4] The representation of onsite green assumes that the hydrogen is produced synchronously with the renewable electricity generation. In this case, electricity is transported between the VRE source and the end user that is producing hydrogen onsite. In contrast, central green hydrogen production is assumed to generate hydrogen at the VRE source (also synchronously), with hydrogen moved to the end user.

| Production technology | Levelized non-energy cost ($2020 / kg of $H_2$) | | Input / Output coefficient (GJ input / GJ hydrogen) | |
|---|---|---|---|---|
| | **2020** | **2050** | **2020** | **2050** |
| Coal + CCS (central) | 1.667 | 1.332 | 1.780 (Coal) | 1.560 (Coal) |
| | | | 0.043 (Electricity) | 0.038 (Electricity) |
| Natural Gas + CCS (central) | 0.779 | 0.746 | 1.367 (Natural Gas) | 1.367 (Natural Gas) |
| | | | 0.020 (Electricity) | 0.020 (Electricity) |
| Natural Gas + CCS (onsite - industrial) | 2.645 | 2.551 | 1.367 (Natural Gas) | 1.367 (Natural Gas) |
| | | | 0.020 (Electricity) | 0.020 (Electricity) |
| Biomass (central) | 1.075 | 0.872 | 2.179 (Biomass) | 2.090 (Biomass) |
| | | | 0.045 (Natural Gas) | 0.025 (Natural Gas) |
| | | | 0.029 (Electricity) | 0.028 (Electricity) |
| Biomass + CCS (central) | 1.770 | 1.414 | 2.606 (Biomass) | 2.297 (Biomass) |
| | | | 0.053 (Natural Gas) | 0.026 (Natural Gas) |
| | | | 0.035 (Electricity) | 0.031 (Electricity) |
| Grid Electrolysis (central) | 1.072 | 0.430 | 1.601 (Electricity) | 1.457 (Electricity) |
| Grid Electrolysis (onsite: industrial & transport) | 1.160 | 0.442 | 1.609 (Electricity) | 1.458 (Electricity) |
| Solar Electrolysis (central) | 5.059 | 1.981 | 1.601 (Electricity) | 1.457 (Electricity) |
| Solar Electrolysis (onsite: industrial & transport) | 5.578 | 2.063 | 1.609 (Electricity) | 1.458 (Electricity) |
| Onshore Wind Electrolysis (central) | 2.753 | 1.077 | 1.601 (Electricity) | 1.457 (Electricity) |
| Onshore Wind Electrolysis (onsite: industrial & transport) | 3.023 | 1.117 | 1.609 (Electricity) | 1.458 (Electricity) |
| Nuclear High Temperature Electrolysis (central) | 3.856 | 3.72 | 3.189 (Thermal Energy - For Heat and Electricity) | 3.189 (Thermal Energy - For Heat and Electricity) |

**Table S1. Hydrogen production – costs and energy/feedstock requirements.** Levelized non-energy costs refer to the production technology costs per kg of $H_2$ and do not include the energy/feedstock costs (e.g., electrolysis non-energy costs are the levelized cost of the electrolyzer but do not include the costs of the required electricity input). 'Input / Output' coefficients are the inverse of efficiencies and are presented in lower heating values (LHV). The values presented do not include the costs or energy requirements for hydrogen T&D, nor for refueling, which are included within the model. The renewable-based hydrogen production costs shown above are based on the capacity factors assumed for the USA. For cost and efficiency assumptions for all model years and regions, see the supplementary data file.

Nuclear HTE assumptions are also informed by NREL H2A models, but because there is no 2018 H2A version for this technology, we utilized the 2008 H2A version (version 2.1)[5], the most recent publicly available, and updated the reactor costs using ATB version 2019. The improvement in nuclear HTE costs is also consistent with GCAM's power sector. No improvement in efficiency is assumed. Unlike all other hydrogen production technologies which are allowed to operate after 2020, nuclear HTE is not available until 2030, reflecting the fact that nuclear technologies often require a longer amount of time to construct and to receive governmental approval for operation.

Onsite blue hydrogen is assumed to be available only within industry given the scale required for $CO_2$ capture and sequestration. This technology is not provided by the H2A models but is created by reducing the assumed size of H2A's central gas SMR + CCS technology to 10% of its designed capacity. Additionally, all hydrogen production technologies have defined lifetimes. Maximum lifetimes are taken from the H2A models. Central production technologies have a 40-year lifetime and 30-year half life, while distributed technologies have a lifetime of 20 years and half life of 15 years.

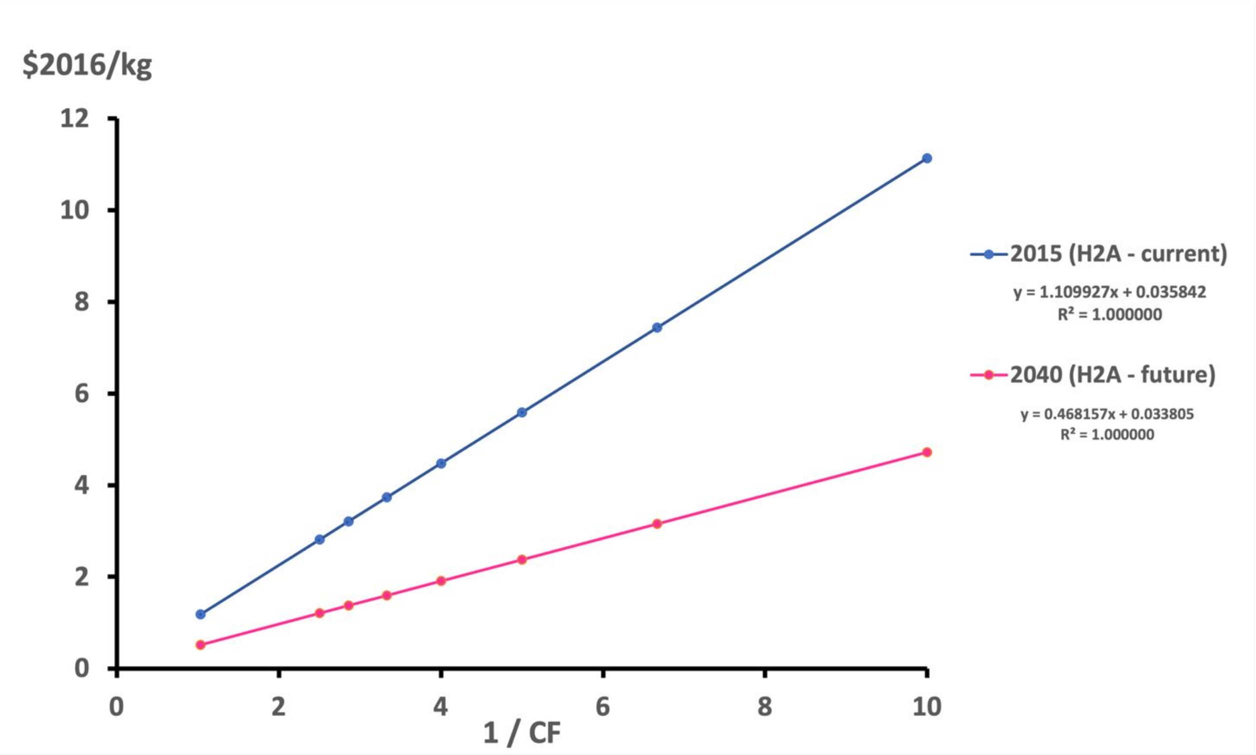


**Figure S1. Levelized electrolyzer non-energy costs as a function of capacity factor for central $H_2$ production.** Based on H2A version 3.2018 central PEM electrolysis production assumptions.

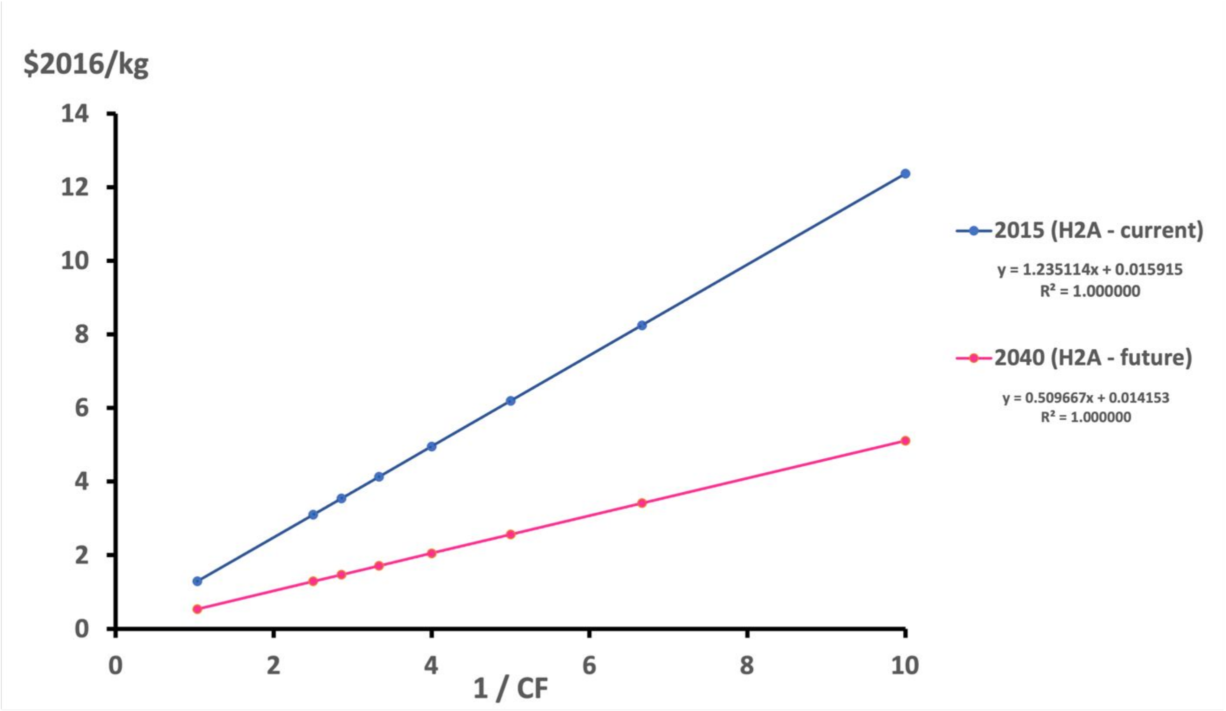


**Figure S2. Levelized electrolyzer non-energy costs as a function of capacity factor for onsite $H_2$ production.** Based on H2A version 3.2018 forecourt PEM electrolysis production assumptions.

In addition to cost and efficiency, technologies in GCAM are calibrated to their historical shares through the "share-weight" parameter. Historically incumbent technologies, such as coal or natural gas, are assigned a unit share-weight of 1, while alternative technologies within a sector have share-weights that reflect whether their historical deployment has been less than or greater than expected given levelized cost competition alone, yielding share-weights that are less than 1 or greater than 1, respectively. Historical share-weights then inform future period energy decisions as past preferences are taken into consideration. This calibration helps avoid unrealistic abrupt fuel-switching in regions and/or sectors where such a change is unlikely, even when considering levelized cost-based competition. However, technologies that have not yet materially deployed have a share-weight of zero in historical years. The speed of a new technology's penetration is determined by the year in which the new technology reaches a share-weight of 1. This represents when the new technology will be fully integrated into the market with its market share solely based on cost.

Hydrogen production technology share-weights influence the competition between technologies within a given subsector. All hydrogen production technologies, other than CCS technologies, are assumed to have a share-weight of 1 beginning in 2025. CCS technologies are assumed to reach 1 in 2030. The CCS share-weight trajectory is consistent with CCS technology share-weights assumed for other GCAM sectors.

***S.1.B. Hydrogen Transmission & Distribution***

In terms of delivering centrally produced hydrogen, we separately represent liquid hydrogen truck and gaseous hydrogen pipeline transmission and distribution. We further include the blending of hydrogen into the natural gas delivery network as a third method of transporting hydrogen to consumers. A wide range of blend limits have been reported as acceptable within the literature. Timmerberg and Kaltischmitt (2019) found that a range from 2-30% had been published and their study assessing the possibility of exporting green hydrogen from Northern Africa to Central Europe via a blend into the existing natural gas infrastructure used 10% hydrogen.[6] The International Energy Agency's 2021 "Net Zero by 2050" study included a blend with a mean of 15% hydrogen globally in 2030,[7] while their 2019 "The Future of Hydrogen" report noted that T&D networks as well as certain end uses (boilers and cooking appliances) could tolerate a blend of 20% or larger.[8] In the High Demand cases, we model a 20% blend by volume, or roughly a 7% blend by energy content.[9] This percentage is within this literature's range and has been previously used as a limit in optimization modeling work by Quarton and Samsatli (2020).[10] We also conservatively phase the blend into natural gas distribution networks over time, given that certain end-use technologies currently installed may not be able to handle a blend of 20% hydrogen, such that all new gas technologies in the relevant end-use sectors are consuming the blend by midcentury (see below).

The levelized non-energy cost and efficiency assumptions for hydrogen delivery, found in *Table S2,* are based on the U.S. DOE's Argonne National Laboratory's Hydrogen Delivery Scenario Analysis Model (HDSAM) version 3.1.[11] Non-energy costs (CAPEX and OPEX) and energy requirements for compression and refrigeration at refueling stations are also included in this study and based on the HDSAM models. Standard HDSAM assumptions were utilized, with modifications made to refueling lifetime (which was set to 20 years). A region selection is required which we defined as Columbus, Ohio given its moderate size and generalizability to other potential district hydrogen markets. The 'H2 Market' type was defined as "Combined Urban/Rural". Liquid hydrogen truck delivery assumptions assumed dispensing at refueling stations via '700 bar gas via pump' and 'Medium' production volume (HDSAM setting 'Mid'). We assumed '700 bar cascade dispensing' and 'High' production volume when generating the gaseous hydrogen pipeline assumptions. Within GCAM, the hydrogen truck representation endogenously selects the drivetrain from the transportation sector's freight road subsector, and therefore HDSAM assumptions are only leveraged for the necessary liquification for onboard storage component of delivery. Total levelized costs (non-energy and energy) for compressing hydrogen in the natural gas pipelines are generated with the same HDSAM assumptions as gaseous pipeline delivery other than production volume which was set to "medium".

Hydrogen delivery from central production facilities is assumed to involve 100 km transmission to the end user, except for renewable-based electrolysis. For central green hydrogen production, we assumed 500 km of transmission since the electrolyzers are assumed to be co-located with large wind or solar farms, which are generally sited where the renewable resource quality is highest, not near hydrogen demand centers. The additional amount of transmission is likewise taken from HDSAM models by adding the additional kilometers required for delivery. The first year for hydrogen pipeline delivery in all cases that allow pipeline delivery is 2030, reflecting that it would take multiple years to build out the pipeline network. We represent the cost of moving electricity for our onsite green $H_2$ production technologies by multiplying the electricity energy requirement to produce a GJ of electrolysis-based hydrogen by the GCAM power sector's assumptions for delivering electricity to the industrial and transportation sectors. The cost of delivering electricity to the transportation sector is higher than delivery to the industrial sector, which is why their costs are larger in *Table S2.*

| | Levelized Cost ($2020 / kg of $H_2$) | | | Input / Output Coefficient (GJ in / GJ hydrogen) | | |
|---|---|---|---|---|---|---|
| **Distribution Method** | **Cost Component** | **2020** | **2050** | **Input** | **2020** | **2050** |
| $H_2$ Liquid Truck | $H_2$ Liquification for onboard storage | 1.893 | 1.744 | Electricity (Liquification / compression for T&D) | 0.277 | 0.225 |
| $H_2$ Gaseous Pipeline | Transmission & Distribution | 0.918* (2030) | 0.845 | Electricity (Compression for T&D) | 0.017* (2030) | 0.017 |
| Central Green $H_2$ 400km additional transmission | $H_2$ liquid truck (Total cost = non-energy + energy) | 0.311 | 0.281 | NA | NA | NA |
| | $H_2$ gaseous pipeline (Total cost = non-energy + energy) | Not Available | 0.617 | NA | NA | NA |
| Onsite Green $H_2$ delivered electricity | Industry (electricity delivery total cost) | 0.485 | 0.385 | NA | NA | NA |
| | Transportation (electricity delivery total cost) | 1.950 | 1.552 | NA | NA | NA |
| Dispensing (transportation) | Compression & storage (Liquid $H_2$ truck delivery) | 1.520 | 1.475 | Electricity (Compression & refrigeration - liquid $H_2$ truck delivery) | 0.016 | 0.016 |
| | Compression & storage ($H_2$ pipeline delivery & onsite production) | 2.374 | 2.228 | Electricity (Compression & refrigeration - $H_2$ pipeline delivery & onsite production) | 0.149 | 0.149 |
| | Retail dispensing additional T&D (Road transportation) | 0.572 | 0.572 | NA | NA | NA |
| Natural Gas + Hydrogen Blend | Compression | 0.067 | 0.061 | Hydrogen | 0.070 | 0.070 |
| | | | | Natural Gas | 0.930 | 0.930 |

**Table S2. Hydrogen transmission, distribution, and refueling - costs and energy/feedstock requirements.** Costs are levelized non-energy costs unless otherwise stated. 'Input / Output' coefficients are the inverse of efficiencies and are presented in lower heating values (LHV). Values are rounded to three decimal places. All central production technologies assume 100 km of hydrogen transmission, other than green hydrogen which requires 500 km of transmission. Zero leakage of hydrogen is assumed. Natural gas pipeline costs are not altered to accommodate the 20% hydrogen blend (by volume), but costs for hydrogen compression into natural gas pipelines is included within the model. *Gaseous pipeline $H_2$ T&D is not available until 2030, so values are presented for this year rather than 2020.

We set improvement rates to allow for reductions in non-energy costs for hydrogen delivery and dispensing, given that HDSAM does not include cost variation overtime. For hydrogen delivery by

gaseous pipeline, liquification for onboard storage in hydrogen truck T&D, and compression of hydrogen for blending into natural gas delivery networks we assumed a 25% total reduction in non-energy costs by 2100. For refueling centers the assumed non-energy cost decrease is differentiated by the type of hydrogen delivery and location of hydrogen production: 20% non-energy cost improvement by 2100 is assumed for refueling stations with gaseous pipeline delivery or onsite production of hydrogen, while only 10% improvement in cost is assumed for refueling stations with hydrogen delivered in liquid trucks. See *Figure S3* for an example of the levelized cost of hydrogen faced by end-users for select hydrogen production technologies. When delivered hydrogen is available liquid hydrogen trucks have a share-weight value of 1 beginning in 2025 while gaseous hydrogen pipelines have a value of 1 beginning in 2030 (and zero beforehand).

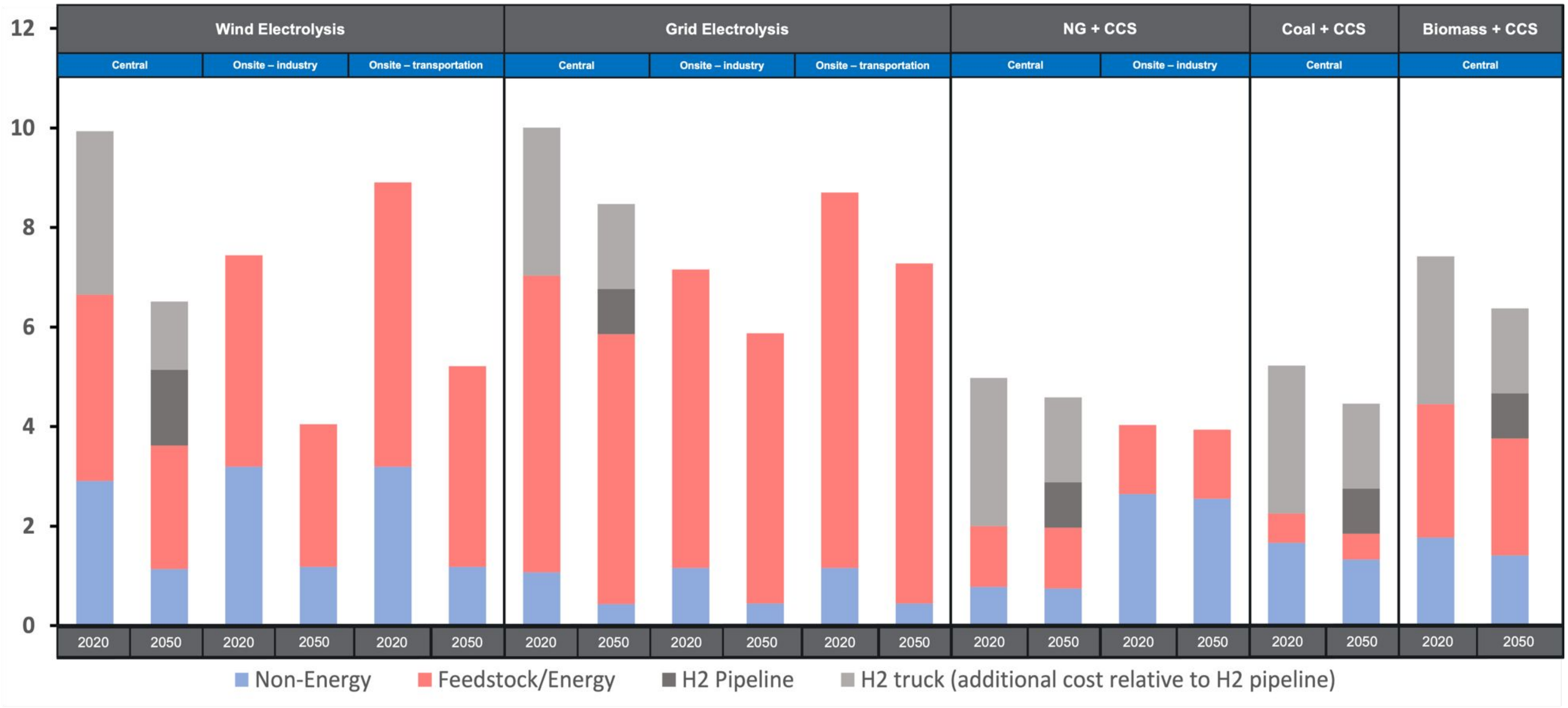


**Figure S3. Levelized cost of hydrogen (LCOH) in $2020 per kg of $H_2$.** The LCOH for a given production method varies regionally and across scenarios due to differences in the projected prices of delivered energy as well as renewable capacity factors. LCOH values presented here use illustrative delivered energy prices and renewable capacity factors which are within the model's ranges for these assumptions and projections across regions. Electricity delivered to centralized and industrial grid electrolysis is assumed to cost $31/GJ, while electricity delivered to onsite production in the transportation sector is assumed to cost $39/GJ. Natural gas is assumed to cost $7/GJ for central production facilities and $8/GJ when delivered to onsite industrial production. Biomass is assumed to cost $8/GJ. Wind-based electrolysis assumes a 35% capacity factor. "Non-Energy" costs refer to levelized capital and operating expenses. "Feedstock/Energy" costs refer to the costs associated with the feedstock and other energy inputs. The "Feedstock/Energy" cost for electrolysis is primarily the cost of electricity, which is either purchased (e.g., grid electrolysis) or produced onsite (e.g., central wind electrolysis). The total cost of delivering hydrogen via trucks is equal to the sum of costs for "$H_2$ pipeline" and "$H_2$ truck (additional cost relative to $H_2$ pipeline)". See the supplementary data file for renewable electrolysis assumptions for all regions and green $H_2$ technologies.

### *S.1.C. End-use sector model modifications*

Gas-fueled technologies in GCAM's industrial and building sectors are allowed to consume the hydrogen and natural gas blend in 'High Demand' scenarios. These technologies include process heat for cement, other industrial energy use (including cogeneration), building heating, and other building needs not including cooling or energy for water supply (e.g., water heating and cooking). District heat production also was modified to allow for blend consumption. 'High Demand' scenarios also allowed for a 25% cost reduction in hydrogen specific industrial technologies (industrial energy use, industrial cogeneration, process heat for cement), with the reduction achieved between 2020 and 2050.[A]

This analysis also included substantial updates to the transportation sector relative to GCAM v5.4, and these updates are now included within the most recent publicly available version of the model (GCAM v6.0).[12] Full information about the assumptions for the transportation sector can be found on JGCRI's GCAM documentation GitHub repository.[B] In our 'Reference Demand' scenarios we hold the capital cost of hydrogen-based modes of transportation flat once they are available. In our 'High Demand' cases we allow for cost improvement in these technologies after the first year of availability, with the improvement aligned with the standard cost trajectory assumptions in GCAM v6.0. In end use sectors, share-weights incorporate various non-cost factors related to technologies (e.g., consumer perception of a new technology, ease of permitting, infrastructure availability, other performance attributes, etc.). 'Year of first availability' as well as the year when hydrogen-powered modes of transport compete with incumbent transportation technologies solely on a basis of levelized costs (i.e., when their technology share-weights reach 1) can be found in *Table S3*. All scenarios allow for cost improvement beyond the first year of availability for non-hydrogen powered transportation.

Industrial final energy hydrogen applications attain share-weights of 1 in 2100. While we explored other alternatives for industry, we elected to maintain our default assumption given the aggregate representation of the industrial sector in this version of GCAM. Alternative assumptions about industry could be explored more easily in a version of GCAM with greater industrial disaggregation, which was not available in the core model at the time that this work commenced. While converging the industrial share-weight sooner would likely increase nearer-term deployment of hydrogen within industry, the significant role for hydrogen within industry is already apparent in the core runs.

---

[A] For industrial cogeneration from hydrogen, this 25% reduction is only applied to the portion of the technology which is hydrogen specific (i.e., the hydrogen combustion portion of the technology's non-energy costs, rather than the portion of non-energy costs which involves the electricity generation).
[B] Documentation for the transportation sector can be found here: https://jgcri.github.io/gcam-doc/cmp/359-Hydrogen_and_transportation.pdf

| GCAM Mode of Transport | Year of First Availability | Year when Share-weight Reaches 1 |
|---|---|---|
| Road-based transport | | |
| Minicar | | 2030 |
| Car | | 2030 |
| Large Car and Truck | | 2030 |
| Bus | 2025 | 2050 |
| Light Truck | | 2050 |
| Medium Truck | | 2050 |
| Heavy Truck | | 2050 |
| Freight Rail | 2025 | 2050 |
| Marine transport | | |
| Domestic Ship | 2035 | 2075 |
| International Ship | | |

**Table S3. GCAM hydrogen-based modes of transport – first year of availability and year when technological share-weight reaches 1.** First year of availability corresponds to the first model period in which the given hydrogen-powered mode of transport is available in GCAM (for this study as well as GCAM v6.0). Year when technological share-weight reaches 1 corresponds to the year when competition between hydrogen-powered modes of transport compete with incumbent transportation technologies solely on a basis of levelized costs.

## S.2. Additional results for the core scenarios

We find that the hydrogen production mix varies depending on whether it is used to meet industrial and transportation demand (*Figure S4*), due in part to the assumption that onsite blue hydrogen is not available in transportation. Onsite green hydrogen provides 44-92% of total hydrogen demand in the transportation sector, and any hydrogen demand not fulfilled by onsite green hydrogen is mostly met by central production when available. In contrast, for the industrial sector onsite green hydrogen provides 34-62% of total production, whereas onsite blue hydrogen provides 19-37% of production (or 29-62% and 16-37% including the hydrogen blended with gas, respectively).

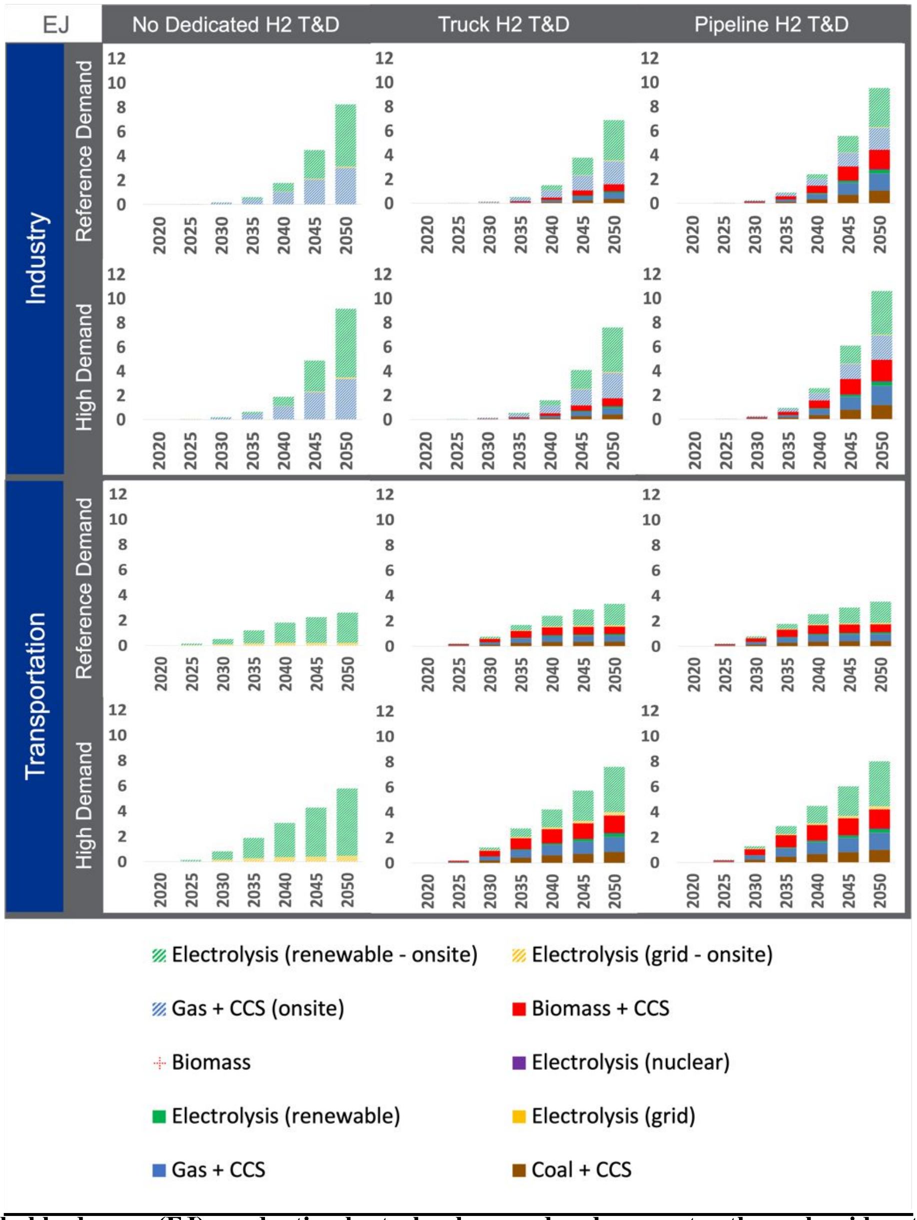


**Figure S4. Global hydrogen (EJ) production by technology and end-use sector through midcentury for the core scenarios.** Centrally produced hydrogen is shown using solid colors, whereas onsite production is shown using diagonal hashed colors. Figure does not include hydrogen blended with natural gas for industrial energy use.

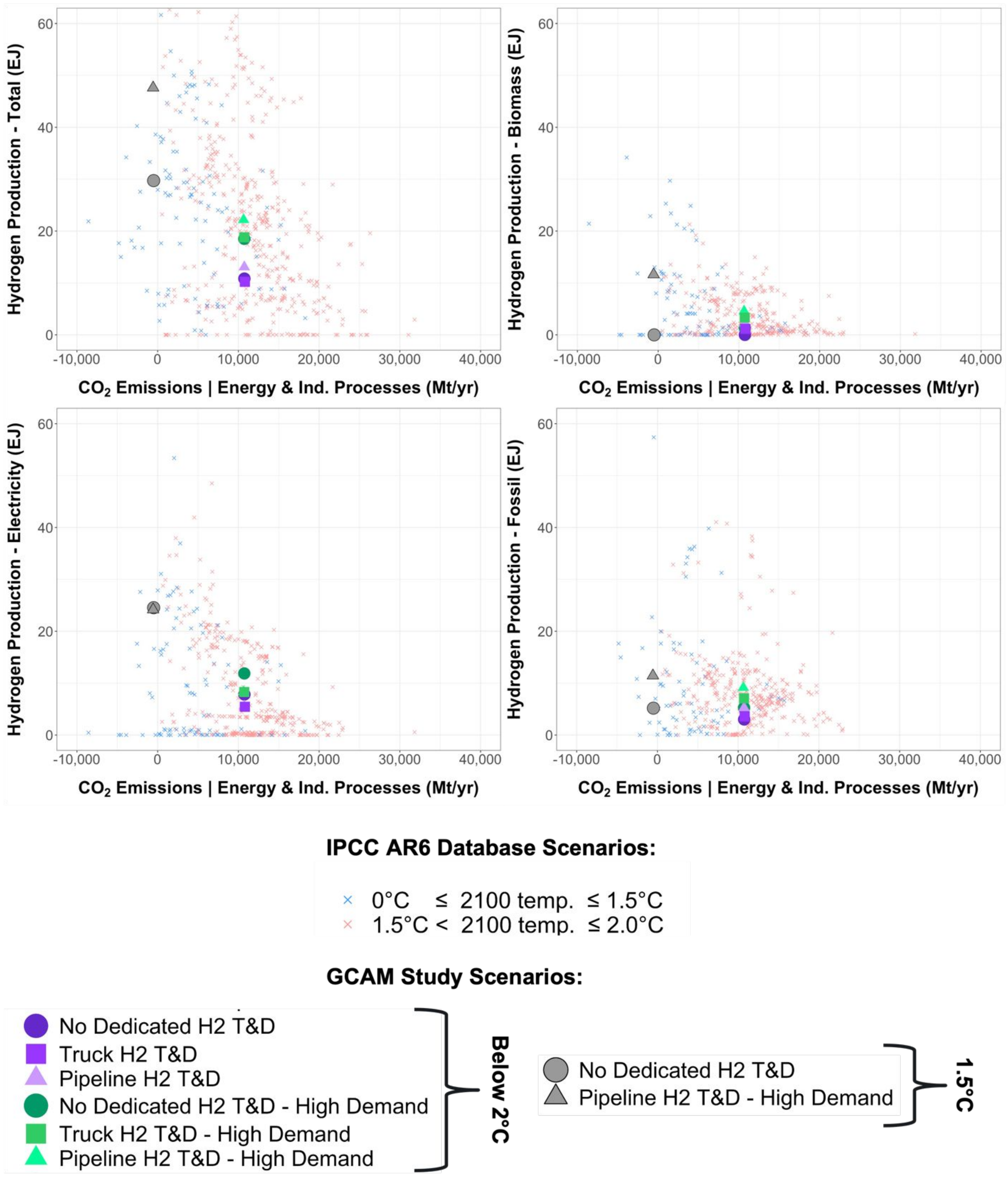


**Figure S5. Global hydrogen production (EJ) in 2050 – core scenarios and 1.5°C cases vs. IPCC AR6 Scenarios.** "Reference Demand" scenarios from this study are portrayed in shades of purple, while "High Demand" scenarios are portrayed in shades of green (however, the 1.5°C sensitivities are all portrayed in grey). Study scenarios are depicted as circles for "No Dedicated $H_2$ T&D", squares for "Truck $H_2$ T&D", and triangles for "Pipeline $H_2$ T&D". IPCC AR6 database scenarios[13] are sorted by their temperature in 2100 for the variable "AR6 climate diagnostics|Surface Temperature (GSAT)|MAGICCv7.5.3|67.0th Percentile". IPCC AR6 scenarios which limit warming to ≤ 1.5°C are depicted in blue circles, while scenarios which exceed this limitation but limit warming to ≤ 2°C are shown as red X's.

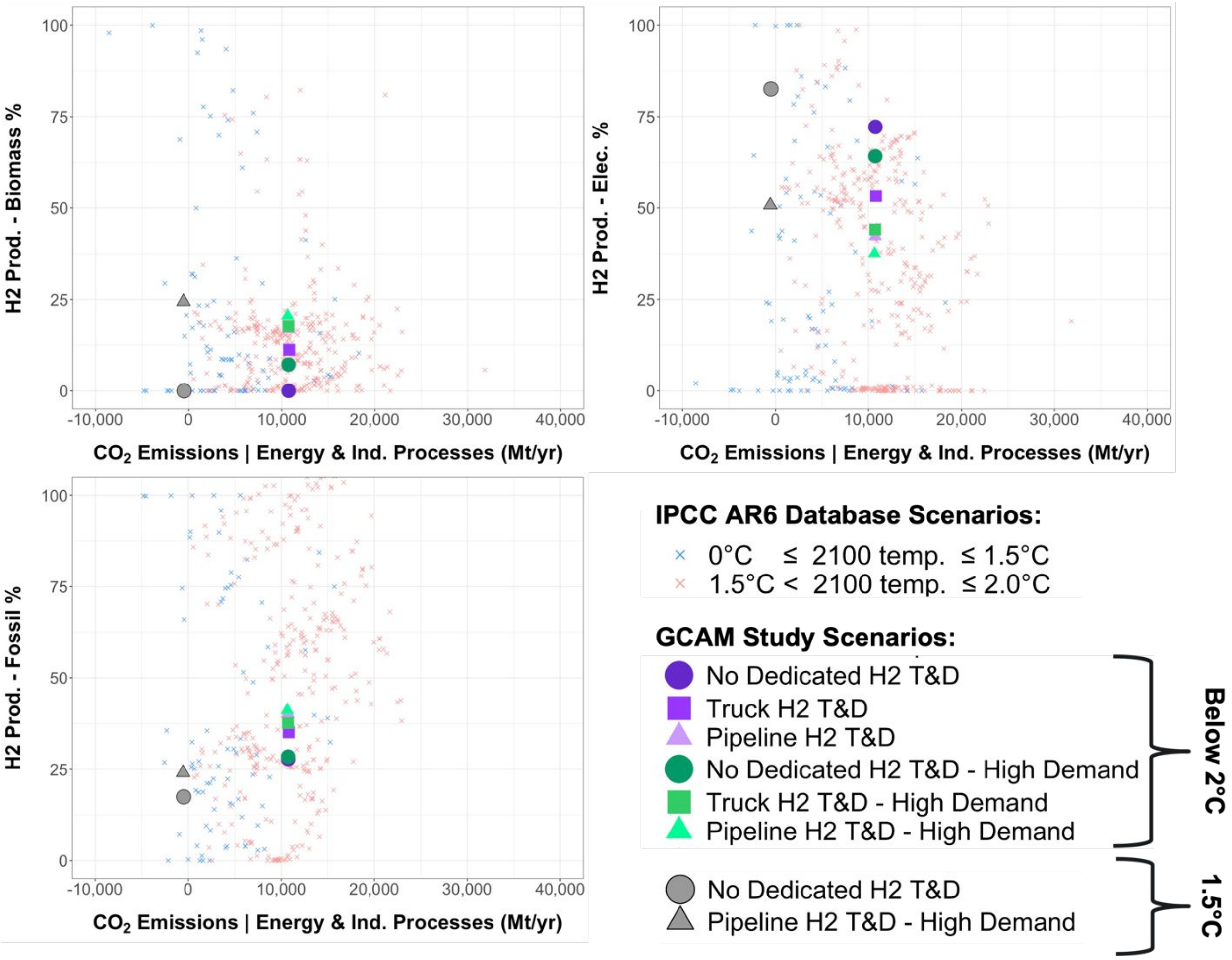


**Figure S6. Percent of global hydrogen production by type in 2050 - main text scenarios and 1.5°C cases vs. IPCC AR6 Scenarios.** “Reference Demand” scenarios from this study are portrayed in shades of purple, while “High Demand” scenarios are portrayed in shades of green (however, the 1.5°C sensitivities are all portrayed in grey). Study scenarios are depicted as circles for “No Dedicated $H_2$ T&D", squares for “Truck $H_2$ T&D”, and triangles for “Pipeline $H_2$ T&D”. IPCC AR6 database scenarios[13] are defined by their temperature in 2100 for the variable “AR6 climate diagnostics|Surface Temperature (GSAT)|MAGICCv7.5.3|67.0th Percentile". IPCC AR6 scenarios which limit warming to ≤ 1.5°C are depicted in blue circles, while scenarios which exceed this limitation but limit warming to ≤ 2°C are shown as red X’s.

| | Scenario: | | | | | | | |
|---|---|---|---|---|---|---|---|---|
| | Truck $H_2$ T&D, Reference Demand | | | | Pipeline $H_2$ T&D, Reference Demand | | | |
| **Sector** | Final energy (EJ) | $H_2$ demand (EJ) | % final energy | % service output | Final energy (EJ) | $H_2$ demand (EJ) | % final energy | % service output |
| **Industry** | **278.9** | **6.9** | **2.5%** | **----** | **279.6** | **9.5** | **3.4%** | **----** |
| Cement | 15.3 | 1.0 | 6.3% | ---- | 15.3 | 1.3 | 8.7% | ---- |
| Other industrial energy use | 196.3 | 5.9 | 3.0% | ---- | 196.9 | 8.19 | 4.2% | ---- |
| **Transportation** | **134.8** | **3.4** | **2.5%** | **----** | **134.8** | **3.5** | **2.6%** | **----** |
| Passenger Vehicle | 39.0 | 0.7 | 1.7% | 1.9% | 39.0 | 0.7 | 1.8% | 2.0% |
| Bus | 6.0 | 0.6 | 9.9% | 22.4% | 6.0 | 0.6 | 10.0% | 22.5% |
| Freight Road | 47.6 | 1.0 | 2.2% | 2.6% | 47.5 | 1.1 | 2.4% | 2.8% |
| Freight Rail | 3.9 | 0.8 | 19.1% | 18.2% | 3.9 | 0.8 | 19.4% | 18.5% |
| Domestic Shipping | 2.3 | 0.1 | 4.8% | 4.9% | 2.26 | 0.1 | 5.9% | 6.1% |
| International Shipping | 13.2 | 0.2 | 1.4% | 1.8% | 13.2 | 0.2 | 1.7% | 2.1% |
| **Buildings** | **171.1** | **0** | **0%** | **----** | **171.1** | **0** | **0%** | **----** |
| Heat | 33.2 | 0 | 0% | ---- | 33.2 | 0 | 0% | ---- |
| Other | 121.3 | 0 | 0% | ---- | 121.3 | 0 | 0% | ---- |
| **Total** | **584.8** | **10.2** | **1.7%** | **----** | **585.5** | **13.1** | **2.2%** | **----** |

**Table S4. Global hydrogen consumption (EJ) by end-use sector in 2050 for remaining Reference Demand scenarios (2°C).** Values (EJ and %) are rounded to the nearest tenth if larger than or equal to 0.05 (otherwise values are presented to the nearest hundredth). 'Other industrial energy use' refers to the GCAM sector ‘industrial energy use’. The subsector ‘Other’ within the buildings sector refers to the sum of GCAM’s ‘residential other’ and ‘commercial other’ subsectors. Subsectors will not sum to the sector total given that there are subsectors which do not consume hydrogen and are thus not reported here (e.g., building cooling, aviation, industrial feedstocks, N fertilizer production, and energy for water supply).

| | Scenario: | | | | | | | |
|---|---|---|---|---|---|---|---|---|
| | No Dedicated $H_2$ T&D, High Demand | | | | Truck $H_2$ T&D, High Demand | | | |
| **Sector** | Final energy (EJ) | $H_2$ demand (EJ) | % final energy | % service output | Final energy (EJ) | $H_2$ demand (EJ) | % final energy | % service output |
| **Industry** | **280.6** | **11.1** | **3.9%** | **----** | **280.6** | **9.5** | **3.4%** | **----** |
| Cement | 15.3 | 1.2 | 8.0% | ---- | 15.3 | 1.0 | 6.6% | ---- |
| Other industrial energy use | 197.8 | 9.8 | 5.0% | ---- | 197.8 | 8.51 | 4.3% | ---- |
| **Transportation** | **134.6** | **5.8** | **4.3%** | **----** | **134.2** | **7.6** | **5.7%** | **----** |
| Passenger Vehicle | 38.6 | 2.3 | 6.1% | 6.6% | 38.5 | 2.9 | 7.4% | 6.7% |
| Bus | 6.0 | 0.7 | 10.8% | 24.3% | 6.0 | 0.7 | 11.0% | 24.6% |
| Freight Road | 47.5 | 1.8 | 3.8% | 4.4% | 47.1 | 2.6 | 5.5% | 6.5% |
| Freight Rail | 3.9 | 0.7 | 18.5% | 17.6% | 4.0 | 0.8 | 20.4% | 19.4% |
| Domestic Shipping | 2.3 | 0.05 | 2.1% | 2.2% | 2.3 | 0.2 | 6.8% | 7.1% |
| International Shipping | 13.2 | 0.2 | 1.6% | 1.9% | 13.2 | 0.5 | 3.8% | 4.7% |
| **Buildings** | **171.7** | **1.5** | **0.9%** | **----** | **171.8** | **1.5** | **0.9%** | **----** |
| Heat | 33.4 | 0.9 | 3% | ---- | 33.5 | 0.9 | 3% | ---- |
| Other | 121.6 | 0.6 | 0.5% | ---- | 121.7 | 0.6 | 0.5% | ---- |
| **Total** | **586.9** | **18.3** | **3.1%** | **----** | **586.6** | **18.6** | **3.2%** | **----** |

**Table S5. Global hydrogen consumption (EJ) by end-use sector in 2050 for remaining High Demand scenarios (2°C).**Values (EJ and %) are rounded to the nearest tenth if larger than or equal to 0.05 (otherwise values are presented to the nearest hundredth). 'Other industrial energy use' refers to the GCAM sector ‘industrial energy use’. The subsector ‘Other’ within the buildings sector refers to the sum of GCAM’s ‘residential other’ and ‘commercial other’ subsectors. Subsectors will not sum to the sector total given that there are subsectors which do not consume hydrogen and are thus not reported here (e.g., building cooling, aviation, industrial feedstocks, N fertilizer production, and energy for water supply).

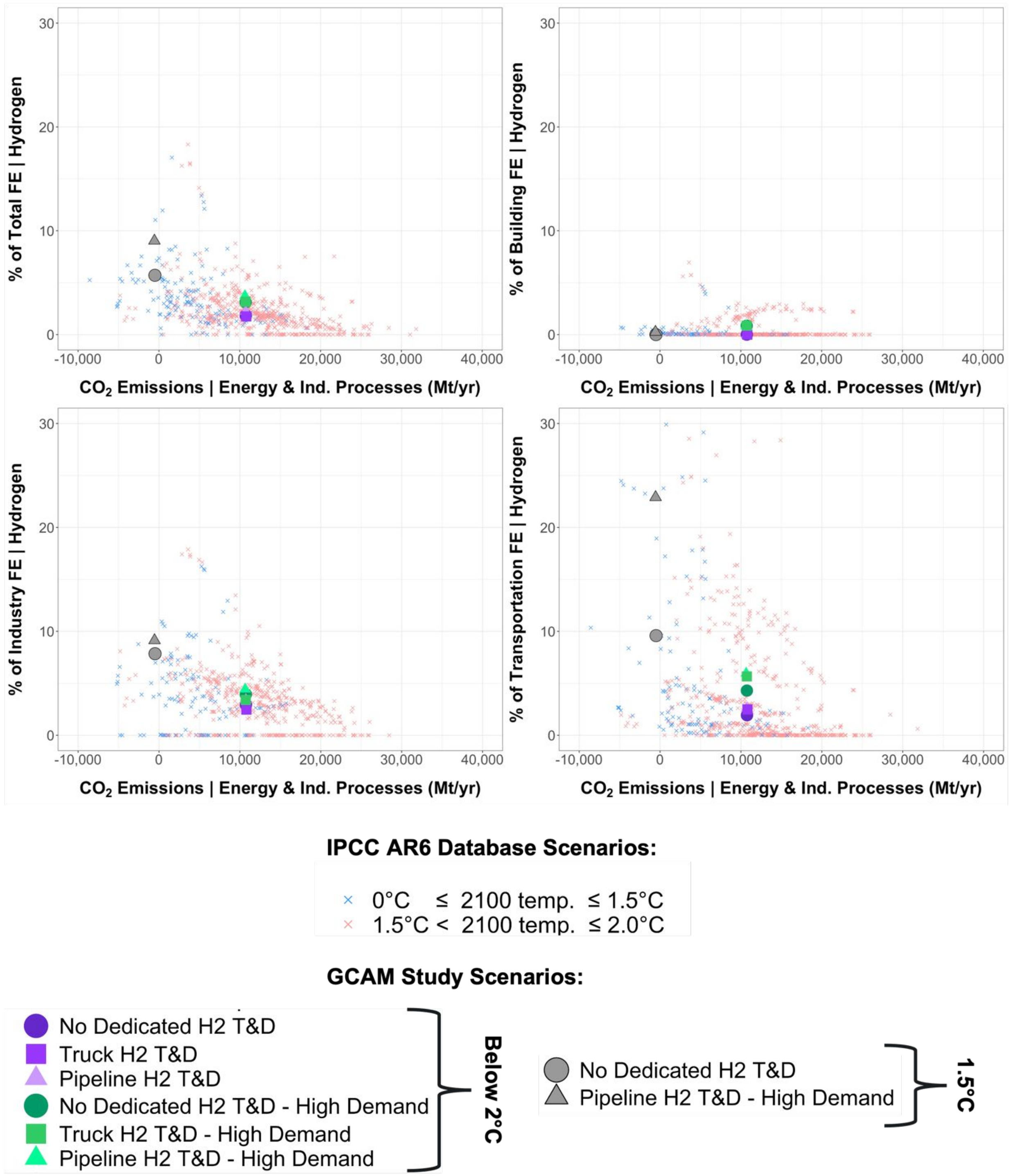


**Figure S7. Percentage of global final energy by type in 2050 – main text scenarios and 1.5°C cases vs. IPCC AR6 scenarios.** "Reference Demand" scenarios from this study are portrayed in shades of purple, while "High Demand" scenarios are portrayed in shades of green (however, the 1.5°C sensitivities are all portrayed in grey). Study scenarios are depicted as circles for "No Dedicated $H_2$ T&D", squares for "Truck $H_2$ T&D", and triangles for "Pipeline $H_2$ T&D". IPCC AR6 database scenarios[13] are defined by their temperature in 2100 for the variable "AR6 climate diagnostics|Surface Temperature (GSAT)|MAGICCv7.5.3|67.0th Percentile". IPCC AR6 scenarios which limit warming to ≤ 1.5°C are depicted in blue circles, while scenarios which exceed this limitation but limit warming to ≤ 2°C are shown as red X's.

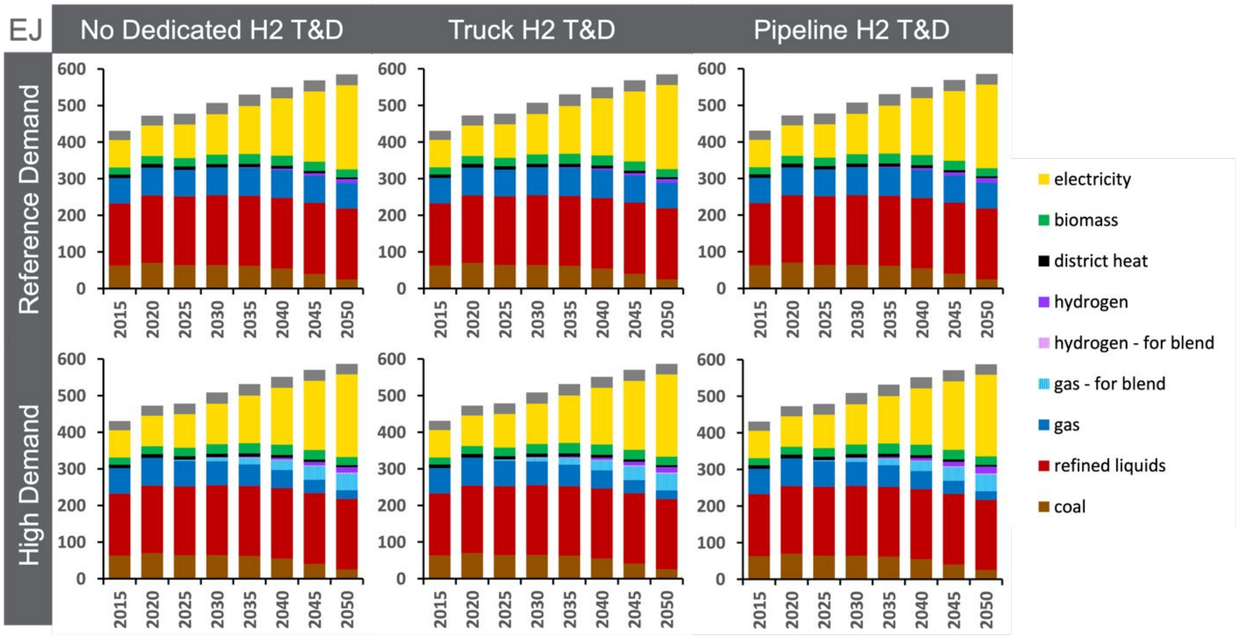


**Figure S8. Total final energy by type in 2050 - core scenarios.**

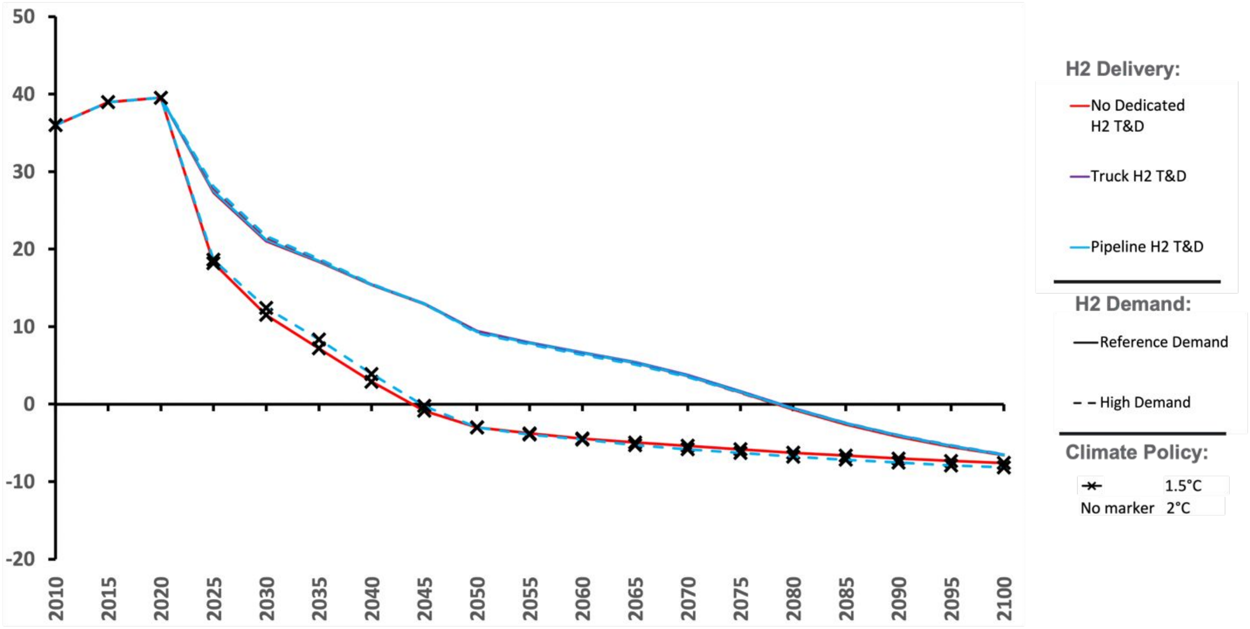


**Figure S9. Global net $CO_2$ emissions over the century (Gt $CO_2$) - core scenarios and 1.5°C cases.** Net $CO_2$ emissions include the net emissions from energy and industrial processes as well as land use change.

| Scenario | | 2025 | 2050 |
|---|---|---|---|
| Below 2°C | No Dedicated $H_2$ T&D | 150 | 314 |
| | Truck $H_2$ T&D | 148 | 310 |
| | Pipeline $H_2$ T&D | 147 | 308 |
| | No Dedicated $H_2$ T&D, High Demand | 144 | 301 |
| | Truck $H_2$ T&D, High Demand | 142 | 297 |
| | Pipeline $H_2$ T&D, High Demand | 141 | 295 |
| 1.5°C | No Dedicated $H_2$ T&D | 680 | 1424 |
| | Pipeline $H_2$ T&D, High Demand | 608 | 1274 |

**Table S6. Carbon prices (2020$ / t $CO_2$) - core scenarios and 1.5°C cases.** Carbon pricing starts in 2025 and rises 3% annually. Land use change emissions face 10% of this price. Values are rounded to the nearest dollar.

*S.2.A. Hydrogen in the second half of the century – below 2°C*

Given the relatively modest uptake of hydrogen by midcentury seen in the core scenarios, we extended the analysis to 2100 to investigate how hydrogen's role may increase over the longer run. The results are inherently more uncertain. Nevertheless, we see important trends. In particular, onsite production of $H_2$ by renewable electrolysis grows, accounting for a minimum 64% by 2100 across all core scenarios (*Figure S10*). This reduces the demand for central production and by the end of the century, scenarios with dedicated hydrogen delivery infrastructure see central production facilities produce at most 28% of total hydrogen globally. As with the near term, we find no substantial difference in total hydrogen supply between the cases where pipelines are available and when no dedicated hydrogen T&D infrastructure exists in the long run (under the same demand assumptions). The High Demand assumptions do increase hydrogen supply, but they also largely maintain the existing proportional mix of production.

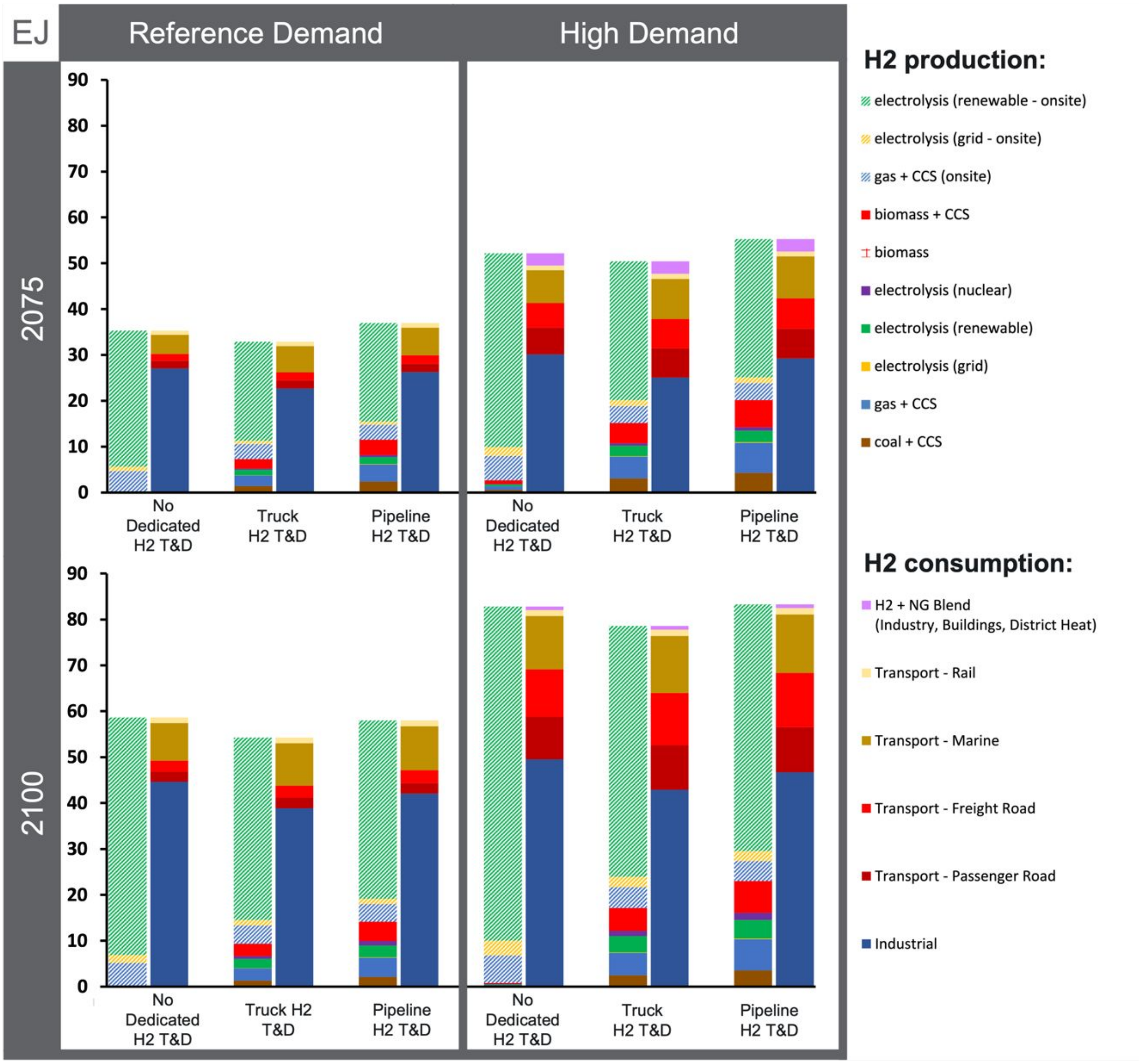


**Figure S10. Global hydrogen production and consumption (EJ) in the long run.** Hydrogen production (left stacked bar) and hydrogen consumption (right stacked bar) in 2075 and 2100 for the core scenarios which limit warming below 2°C. Centrally produced hydrogen is shown using solid colors, whereas onsite production is shown using diagonal hashed colors.

In the second half of the century hydrogen's role as a final energy carrier is still modest, meeting just 8 to 12% of total final energy demand in 2100 (54-83 EJ of $H_2$ consumed). But this represents an expanded contribution to decarbonization relative to midcentury. The industrial sector remains the largest user of hydrogen in the long run, consuming 39- 50 EJ of hydrogen in 2100 (55-76% of total hydrogen consumption, not including $H_2$ consumed in the form of natural gas/$H_2$ blends). While not the dominant provider of final energy for GCAM's industrial energy use sector, hydrogen plays a notable role satiating between 13 and 18% of industrial energy use demand. In fact, by 2100 hydrogen could provide 79% of energy demand for cement without dedicated hydrogen delivery infrastructure and with Reference Demand cost assumptions. For the transportation sector, we find a larger range of potential final energy coming from $H_2$ across the scenarios (8-20% in 2100). While hydrogen provides 4-20% of final energy for freight trucks by the end of the century, the energy carrier plays a larger role in marine applications. Even with conservative cost assumptions hydrogen provides 65-73% of final energy for international shipping in 2100. Lastly, consistent with the diminished role blended hydrogen and natural gas saw moving from 2030 to 2050, by 2100 hydrogen consumption coming from the blend is found to be minor (1%).

### S.3. ***Sensitivity: limiting warming to 1.5°C***

In this section we examine hydrogen production and consumption under scenarios that limit warming to 1.5°C for our two 'bookend' scenarios (*Figure S11*). In these scenarios, onsite green hydrogen remains the dominant method of production, providing an even larger share of total production compared to the corresponding 2°C scenarios (45-81% vs. 32-69% in 2050). The percentage and absolute amount of hydrogen production coming from BECCS is also larger when pipeline delivery is available compared to the corresponding 2°C scenarios (11.6 EJ or 24% in 2050 vs. 4.6 EJ or 21%). This is primarily driven by the increased subsidy for BECCS under higher carbon prices (more than 4 times larger in 2050 in the 1.5°C scenarios than in the 2°C scenarios). In addition, hydrogen production accounts for a larger share of total biomass consumption (25% under 1.5°C as compared to 7% under 2°C) for the same scenario and year. And while the absolute amount of blue hydrogen production increases by 2-3 EJ in the 1.5°C scenarios compared to the corresponding 2°C scenarios, the share of blue hydrogen is lower by 9-10 percentage points. These results are not surprising, as higher carbon prices will favor hydrogen production technologies with a zero or negative carbon intensity.

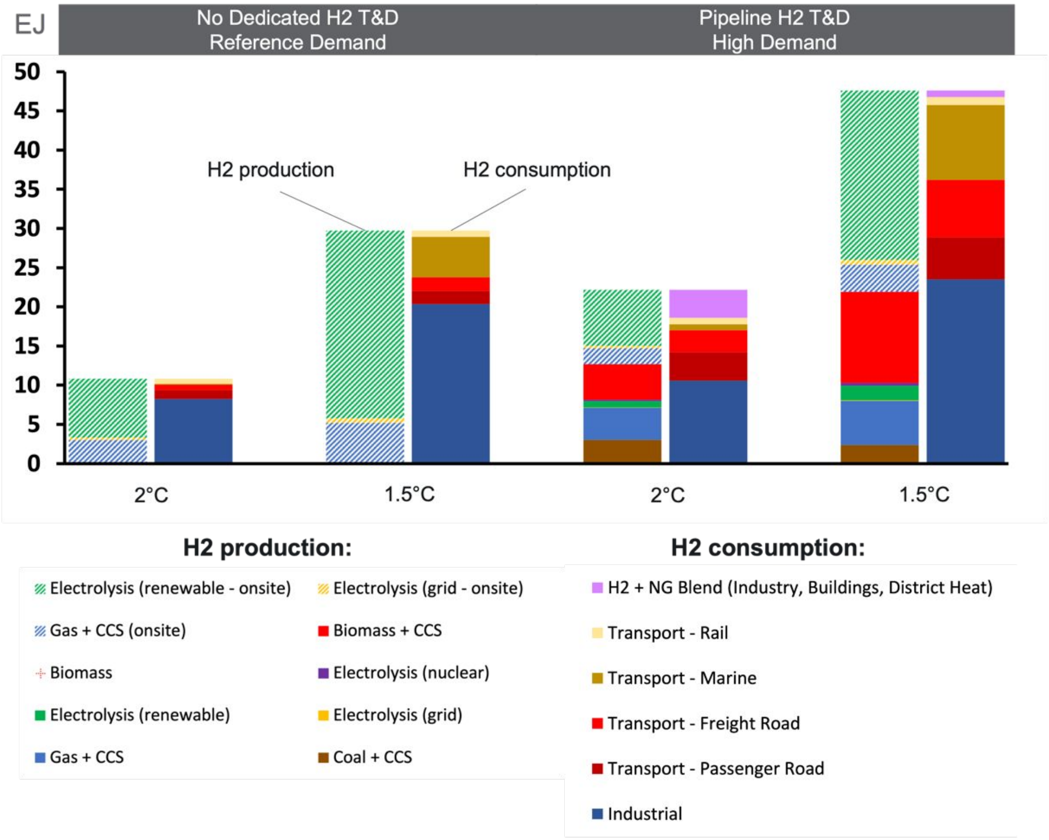


**Figure S11. Hydrogen production and consumption (EJ) in 2050 for two of the core cases for two different climate objectives (1.5°C and 2°C).** Centrally produced hydrogen is shown with solid colors, whereas onsite production is shown as diagonal hashed colors.

Total hydrogen consumption in the 1.5°C scenarios (30-48 EJ) is more than twice the amount in the corresponding 2°C scenarios (10-22 EJ) in 2050, as traditional energy carriers are replaced more rapidly with zero-carbon energy carriers. In fact, by midcentury the percentage of total final energy from hydrogen increases from roughly 2-4% in the 2°C scenarios to 6-9% in the 1.5°C scenarios. Hydrogen

accounts for 10-23% of transportation final energy in the 1.5°C scenarios, which is a substantial increase over the share attained in the 2°C scenarios (2-6%). In fact, consumption from the transportation sector is only slightly smaller (<1 EJ) than that of the industrial sector in the 1.5°C High Demand scenario with pipeline T&D available. Most notably, hydrogen consumption for marine transport under 1.5°C (5.1-9.6 EJ) is now larger than hydrogen consumption for passenger road or freight road. While hydrogen accounts for 48-80% of marine final energy in 2050, this is primarily hydrogen deployed in international shipping (5-9 EJ) where it accounts for 58-90% of final energy.

| | **Scenario:** | | | | | | | |
|---|---|---|---|---|---|---|---|---|
| | **No Dedicated $H_2$ T&D, Reference Demand, 1.5°C** | | | | **Pipeline $H_2$ T&D, High Demand, 1.5°C** | | | |
| **Sector** | Final energy (EJ) | $H_2$ demand (EJ) | % final energy | % service output | Final energy (EJ) | $H_2$ demand (EJ) | % final energy | % service output |
| **Industry** | **259.4** | **20.4** | **7.9%** | | **261.7** | **23.9** | **9%** | |
| Cement | 14.7 | 8.0 | 54.3% | | 14.8 | 7.7 | 52.3% | |
| Other industrial energy use | 180.1 | 12.4 | 6.9% | | 182.1 | 16.2 | 8.9% | |
| **Transportation** | **97.7** | **9.4** | **9.6%** | | **101.5** | **23.3** | **22.9%** | |
| Passenger Vehicle | 28.1 | 0.9 | 3.3% | 2.5% | 29.0 | 4.6 | 15.7% | 12.7% |
| Bus | 4.6 | 0.7 | 16.0% | 27.9% | 4.7 | 0.8 | 17.0% | 30.1% |
| Freight Road | 35.2 | 1.8 | 5.0% | 4.6% | 35.9 | 7.3 | 20.4% | 20.3% |
| Freight Rail | 3.1 | 0.8 | 25.9% | 21.4% | 3.4 | 1.0 | 29.3% | 24.8% |
| Domestic Shipping | 2.0 | 0.1 | 5.7% | 4.3% | 2.3 | 0.6 | 8.2% | 22.4% |
| International Shipping | 8.7 | 5.0 | 57.7% | 51.0% | 10.0 | 9.0 | 90.5% | 88.2% |
| **Buildings** | **163.5** | **0** | **0%** | | **163.2** | **0.4** | **0.3%** | |
| Heat | 32.9 | 0 | 0% | | 32.2 | 0.3 | 1.0% | |
| Other | 114.9 | 0 | 0% | | 115.3 | 0.1 | 0.1% | |
| **Total** | **520.5** | **29.7** | **5.7%** | | **526.4** | **47.6** | **9.0%** | |

**Table S7. Global hydrogen consumption (EJ) by end-use sector in 2050 for 1.5°C Scenarios.** Values (EJ and %) are rounded to the nearest tenth if larger than or equal to 0.05 (otherwise values are presented to the nearest hundredth). 'Other industrial energy use' refers to the GCAM sector ‘industrial energy use’. The subsector ‘Other’ within the buildings sector refers to the sum of GCAM’s ‘residential other’ and ‘commercial other’ subsectors. Subsectors will not sum to the sector total given that there are subsectors which do not consume hydrogen and are thus not reported here (e.g., building cooling, aviation, industrial feedstocks, N fertilizer production, and energy for water supply).

Industrial final energy from hydrogen in the 1.5°C scenarios is still a small share of total industrial final energy in 2050 (8-9% including industrial feedstocks), but it is larger than in the 2°C scenarios (2-4%). However, 52-54% of final energy for cement (mostly process heat) is generated using hydrogen in the 1.5°C scenarios, compared to 6-9% in the 2°C scenarios. Lastly, in the 1.5°C scenarios, hydrogen blended with natural gas accounts for less than 2% of hydrogen consumption in 2050, compared to 16-19% in the 2°C scenarios. This is because the higher carbon prices in the 1.5°C scenarios more

rapidly drive a transition away from energy carriers that still emit $CO_2$. For example, at midcentury building heating is more heavily electrified by midcentury in the 1.5°C scenarios (19-20% for the 2°C scenarios vs. 42-45% for the 1.5°C cases).

## S.4. Additional sensitivities

### *S.4.A. Sensitivity: biomass and CCS limited (2°C)*

Bioenergy availability could affect hydrogen deployment, both in terms of the production mix (bio gasification for hydrogen production) and in terms of the need for additional carbon free energy carriers - particularly in end uses that may have consumed biomass or bio-derivative products (e.g., bioliquids) if accessible. Additionally, when biomass is coupled with carbon sequestration it can provide a negative source of emissions which would be incentivized by carbon policies. The availability of carbon sequestration also has the potential to impact hydrogen utilization in multiple ways. For one, it could alter the production mix for hydrogen, particularly since any fossil routes to low-carbon hydrogen require CCS. Additionally, limited ability for carbon sequestration could increase the need for zero-carbon energy carriers in end-use sectors that might otherwise rely on removals via CCS to decarbonize.

Given these reasons, we model two additional scenarios: one with limited bioenergy available and a second with limited bioenergy and limited carbon sequestration. 'Bio Low' scenarios here limit primary energy utilization of biomass (other than 'traditional biomass'[C]) to less than 50 EJ per year, in line with the constraint utilized within McJeon et al. (2021).[14] "Bio Low / CCS Low" scenario constrains the amount of globally available carbon sequestration to that of the 25$^{th}$ percentile for scenarios limiting warming to 2°C through the century within the IPCC's AR6 scenario database[13], by year. Scenarios are classified according to their temperature for the following variable within the database: "AR6 climate diagnostics | Surface Temperature (GSAT) | MAGICCv7.5.3 | 67.0th Percentile". *Table S8* displays the limitation by model period.

| Year | 25$^{th}$ Percentile |
|---|---:|
| 2020 | 0 |
| 2025 | 0 |
| 2030 | 194 |
| 2035 | 956 |
| 2040 | 2,001 |
| 2045 | 3,036 |
| 2050 | 4,120 |
| 2055 | 5,332 |
| 2060 | 6,731 |
| 2065 | 7,724 |
| 2070 | 8,612 |
| 2075 | 9,332 |
| 2080 | 9,830 |
| 2085 | 9,938 |
| 2090 | 10,330 |
| 2095 | 10,733 |
| 2100 | 11,017 |

**Table S8. Carbon sequestration limitation (Mt $CO_2$ / yr).** Values are based on reporting for the variable "Carbon Sequestration | CCS" within the IPCC AR6 database and are rounded to the nearest whole number.

[C] Traditional biomass includes non-modern sources of biomaterial such as agricultural residues, animal dung, and fuelwood. This is in line with the IEA's "primary solid biomass" energy resource (or 'product'). More details about traditional biomass within GCAM can be found at http://jgcri.github.io/gcam-doc/supply_energy.html.

Each of these scenarios allows for pipeline delivery of hydrogen and utilizes the Reference Demand set of assumptions. The role of biomass is somewhat sensitive to assumptions about biomass and CCS. Specifically, we find that limiting bioenergy (‘Bio Low’) increases total hydrogen production by 4 EJ and expands the role of green and blue hydrogen (*Figure S12*). In this case, the benefit of hydrogen as an alternative energy carrier exceeds the cost of constraining one of the $H_2$ supply options (BECCS $H_2$). On the other hand, imposing the biomass limitation simultaneously with limited carbon sequestration (the Bio Low + CCS Low scenario) results in roughly the same amount of total hydrogen production, with increased reliance on renewable electrolysis, which then accounts for more than 75% of the production mix. In this scenario, the cost of constraining multiple $H_2$ options balances the benefit of $H_2$ as an alternative energy carrier.

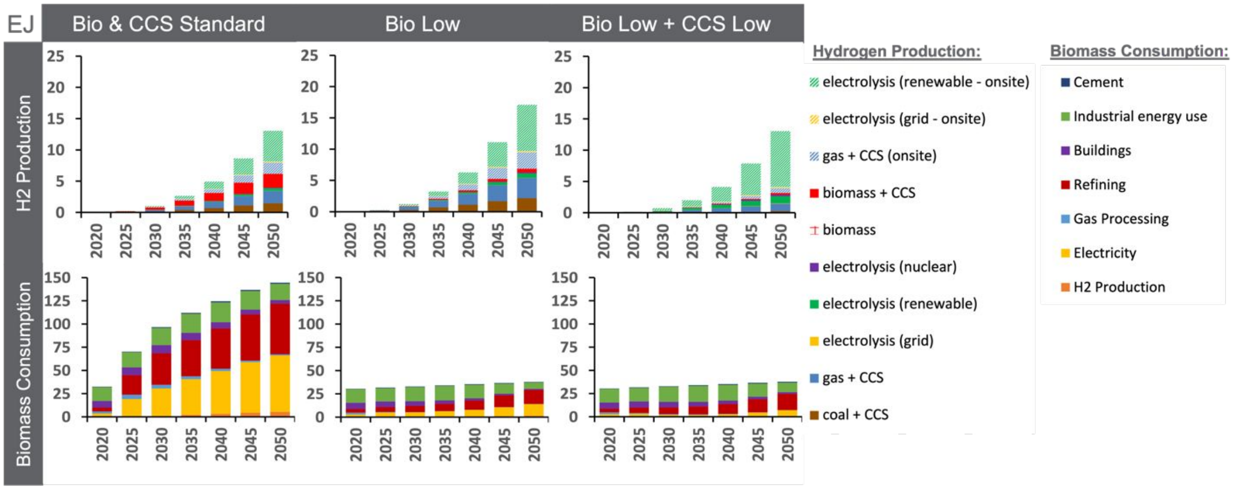


**Figure S12. Global hydrogen production and biomass consumption (EJ).** All scenarios in this figure contain the Reference Demand assumptions and allow for pipeline delivery of hydrogen. The “Bio & CCS Standard” scenario refers to the “Pipeline $H_2$ T&D, Reference Demand” scenario from the standard cases. Centrally produced hydrogen is shown with solid colors, whereas onsite production is shown as diagonal hashed colors.

The broader energy transition assumptions for bioenergy and carbon sequestration availability also impact the consumption of $H_2$ (*Figure S13*). An expansion in hydrogen utilization is found for industry (~3 EJ increase) as well as international shipping (~1 EJ increase) when biomass is limited. While a 1 EJ increase of hydrogen for international shipping may seem trivial, this is equivalent to a rise from 2% to 9% of final energy for this transportation subsector. In terms of industry, a large portion of this gain entails larger deployment of hydrogen for cement process heat - roughly doubling from 1.3 EJ to 2.6 EJ (or from about 9% to about 17% of final energy). The results are perhaps not surprising, given that the combustion of biomass can facilitate industrial process heat demand under decarbonization, and biofuels are also one method of providing a low-carbon fuel for heavy-duty transportation needs. On the other hand, the combined effect of the bioenergy and CCS constraints is not found to impact hydrogen consumption meaningfully.

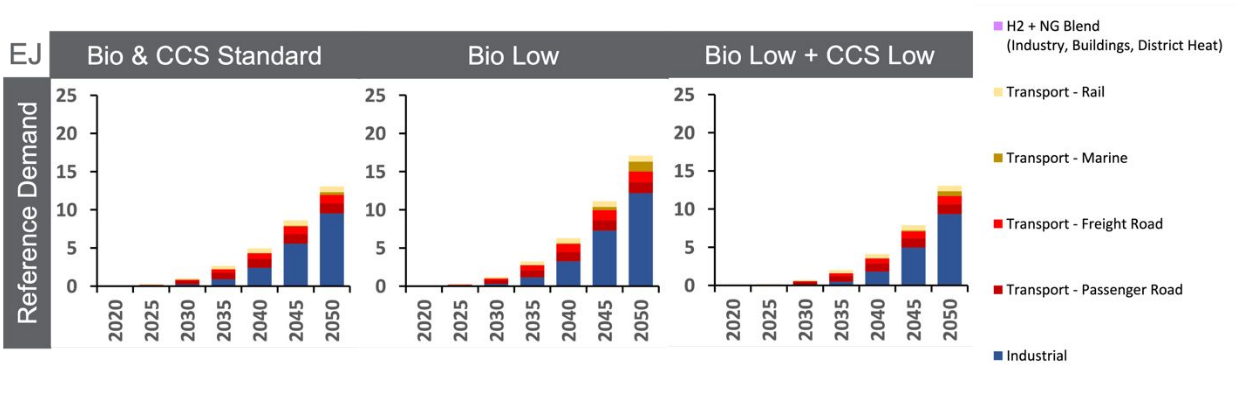


**Figure S13.** **Global hydrogen consumption (EJ) by end-use sector through midcentury for the Bio and CCS Low scenarios.**

### *S.4.B. Sensitivity: no onsite $H_2$ production (2°C)*

Given that our results suggest a large share of hydrogen could be produced onsite, primarily through renewable electrolysis, we conduct two sensitivities where hydrogen supply is restricted to centralized facilities. Results may also help explain outcomes found in other modeling work where onsite production of hydrogen is not considered (i.e., all hydrogen is delivered and faces the associated costs). Scenarios are simulated under reference and high demand assumptions, with warming limited below 2°C in both cases. Hydrogen transmission by truck or pipeline is available in each of these sensitivity cases as well, so we compare to the main text scenarios with the same climate objective and pipeline availability for illustrative purposes.

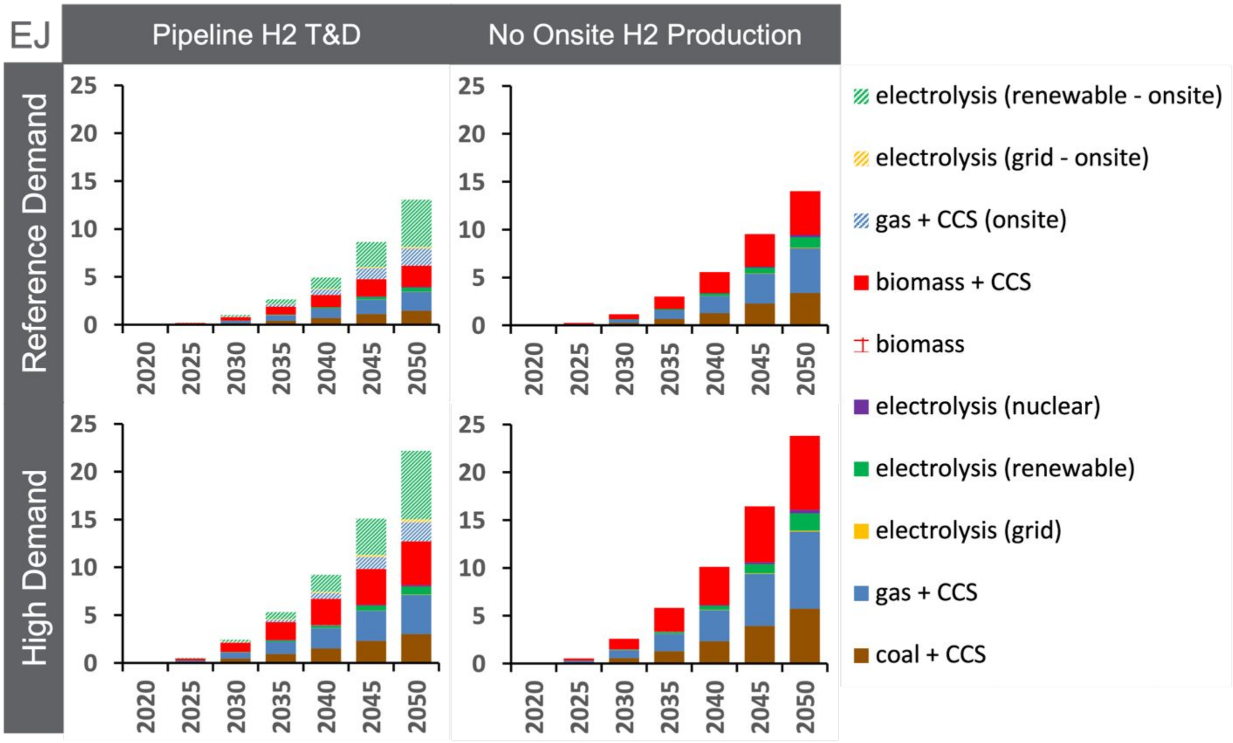


**Figure S14. Global hydrogen production (EJ) - No Onsite $H_2$ Production scenarios.** Centrally produced hydrogen is shown with solid colors, whereas onsite production is shown as diagonal hashed colors.

As seen in *Figure S14*, limiting $H_2$ production to central facilities is not found to alter the scale of total hydrogen production significantly (e.g., < 1 EJ increase under reference demand assumptions). However, the production mix is considerably different. Without the ability to produce hydrogen at the end user green hydrogen is less competitive – decreasing from 36-41% to 8% of total hydrogen supply at midcentury (across paired scenarios). Production is now mainly based on blue hydrogen and BECCS. The latter of these two sees a large increase in its share compared to the paired counterpart scenarios, and now accounts for roughly a third of total hydrogen. When onsite production and pipeline T&D were allowed BECCS only contributed 17-21%. Natural gas with CCS's share of total hydrogen supply also increases slightly (from 28-29% to 33-34%). Coal with CCS hydrogen production also nearly doubles when onsite production is restricted for both sensitivities, compared to their counterparts. In terms of demand, we find that this supply side sensitivity does not meaningfully alter hydrogen consumption (*Figure S15*).

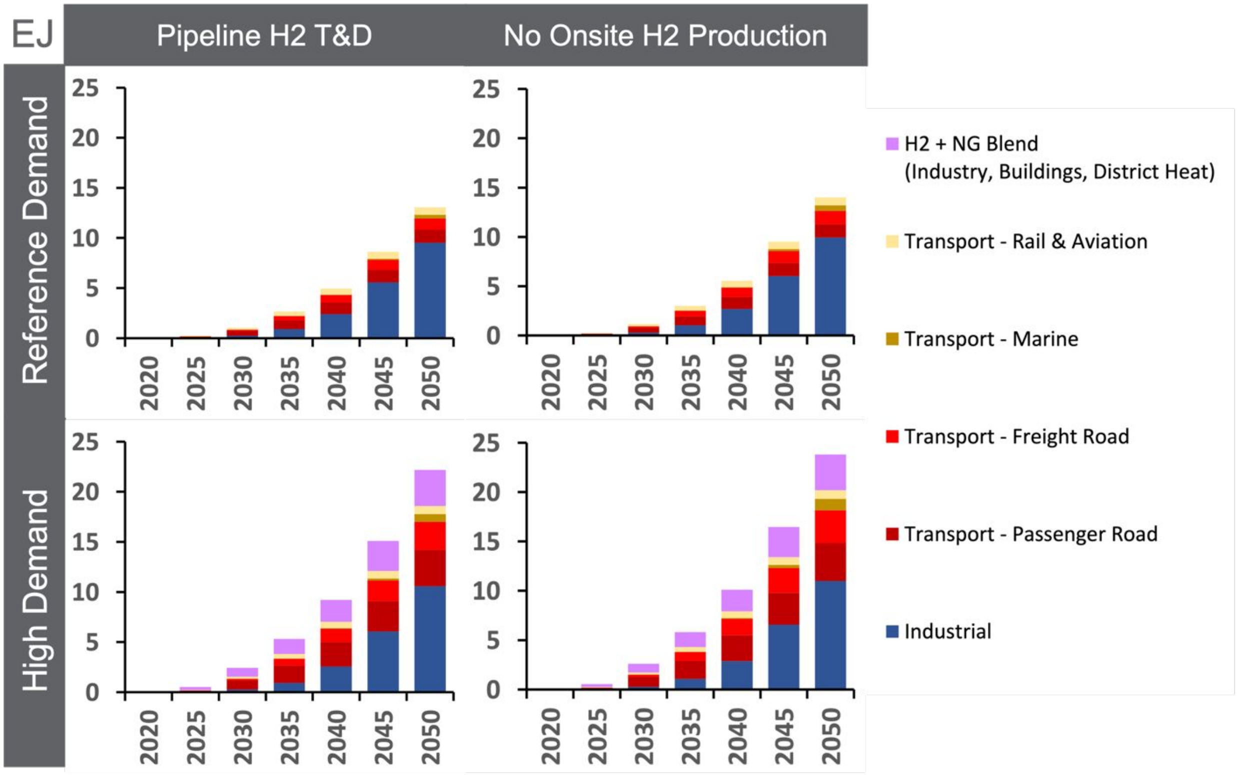


**Figure S15. Global hydrogen consumption by end-use sector (EJ) - No Onsite $H_2$ Production scenarios.** Only the amount of hydrogen within the hydrogen/natural gas blend is included here (pink).

### *S.4.C. Sensitivity: direct air capture (DAC) available (2°C)*

The deployment of facilities that directly capture large amounts of atmospheric $CO_2$ are not considered within our main text scenarios. One could hypothesize that if such technologies were economically competitive with other mitigation strategies - such as the use of low-carbon hydrogen - that they could reduce the need to deploy alternative decarbonization technologies. Given this possibility, we conduct an additional two sensitivities on our main text bookend scenarios which limit warming below 2°C: (1) *No Dedicated $H_2$ T&D*, and (2) *Pipeline $H_2$ T&D, High Demand*.

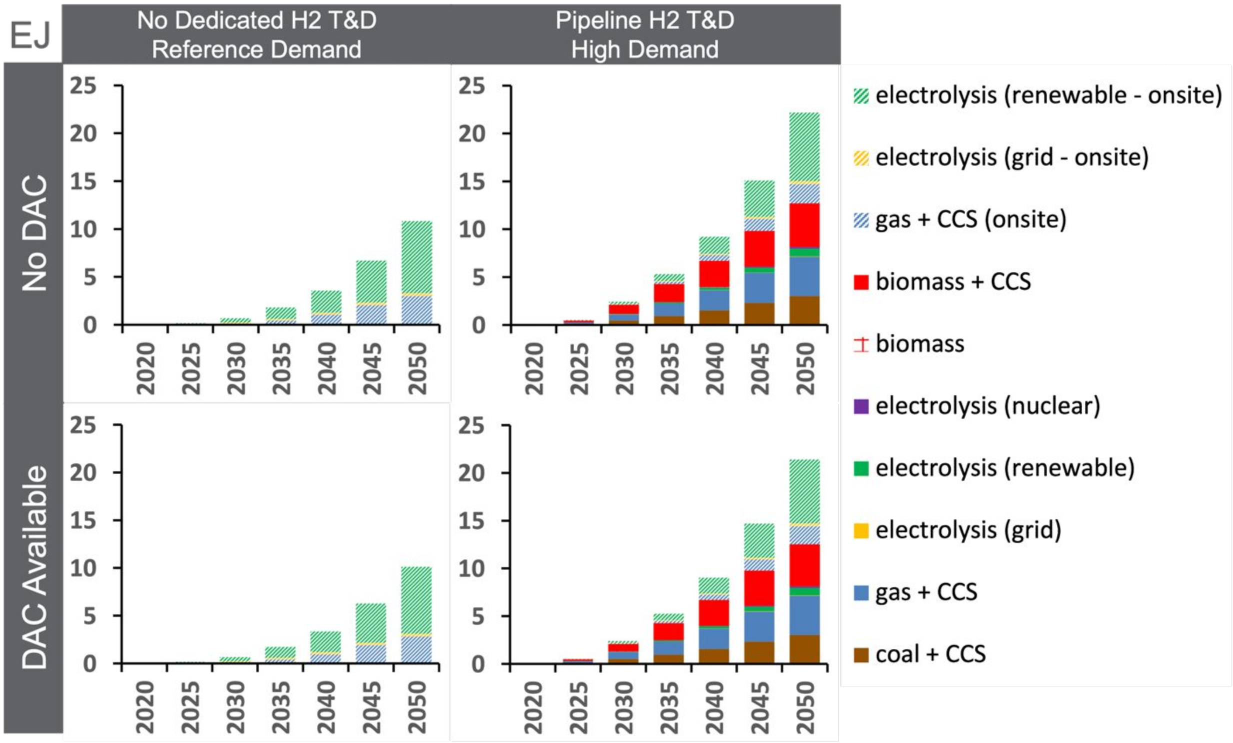


**Figure S16. Global hydrogen production (EJ) - Direct Air Capture (DAC) scenarios.** Centrally produced hydrogen is shown with solid colors, whereas onsite production is shown as diagonal hashed colors.

While not a part of GCAM's default configuration, the model recently included direct air capture (DAC) assumptions for each of the five shared socioeconomic pathways (SSPs). These are detailed in Fuhrman et al (2021).[15] We utilize GCAM's SSP2 DAC assumptions, or the "middle-of-the-road" projections, to analyze how the availability of this negative emissions technology impacts hydrogen deployment. Specifically, three types of DAC are considered: (1) high-temperature DAC via natural gas process heat, (2) high-temperature DAC via grid electricity process heat, and (3) low-temperature DAC via electric heat pump.

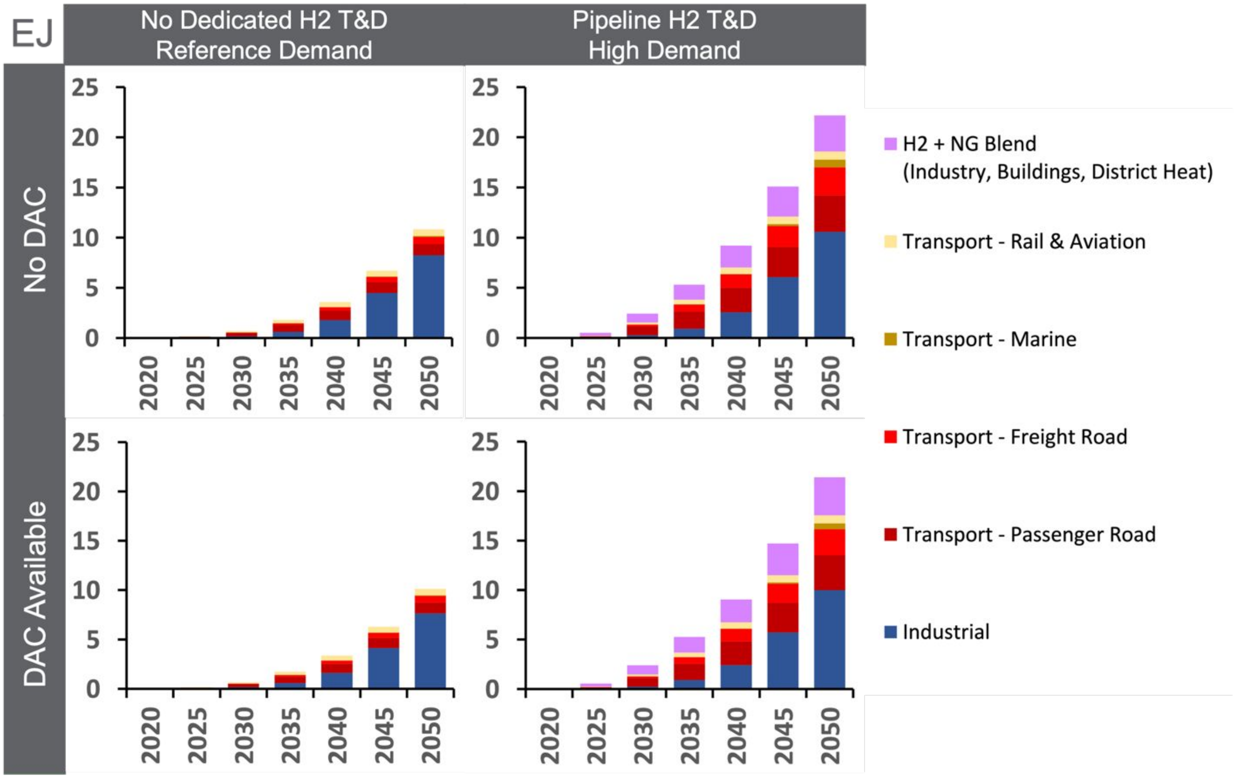


**Figure S17. Global hydrogen consumption by end-use sector (EJ) - Direct Air Capture (DAC) scenarios.** Only the amount of hydrogen within the hydrogen/natural gas blend is included here (pink).

Our results indicate that the availability of DAC doesn't meaningfully alter the scale or mix of hydrogen production (*Figure S16*). $H_2$ consumption is not altered significantly either (*Figure S17*). While these results may change if the ability to couple DAC and $H_2$ production for the creation of synthetic fuels had been considered, it is still worth noting hydrogen is not sensitive to DAC without carbon capture and utilization (CCU). There is one noticeable change in the decarbonization trajectory though, as this negative emissions technology allows the economy to delay early climate change mitigation in favor of deeper net negative emissions in the second half of the century (*Figure S18)*.

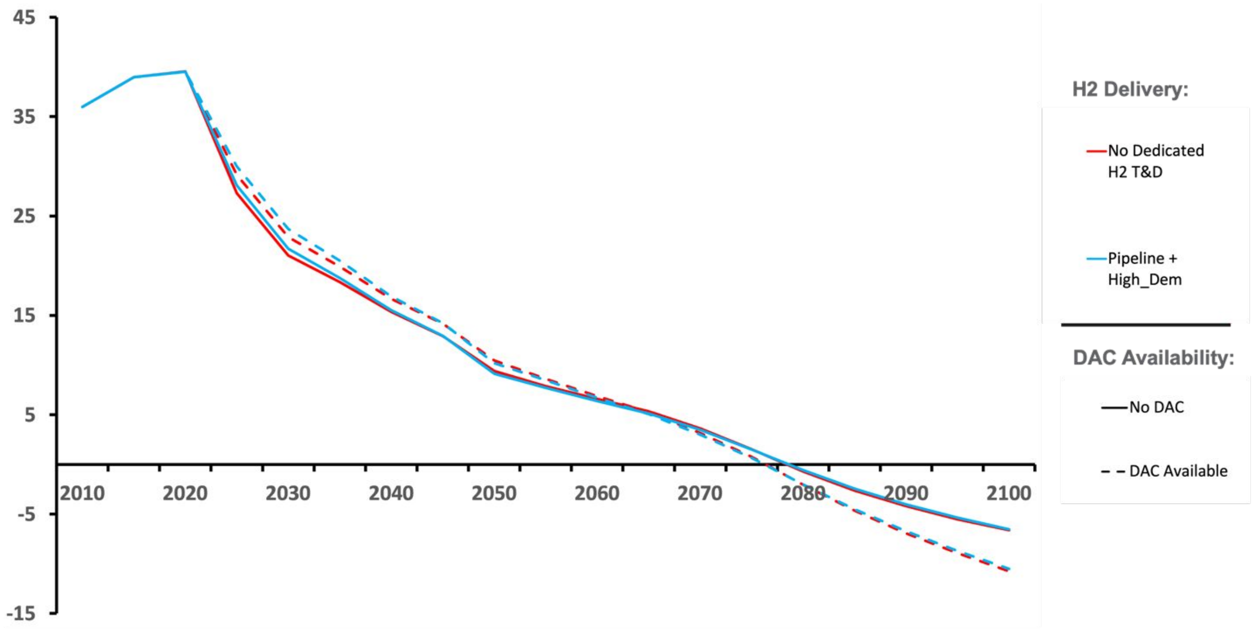


**Figure S18. Global net $CO_2$ emissions over the century (Gt $CO_2$) - Direct Air Capture (DAC) scenarios.** Net $CO_2$ emissions include the net emissions from energy and industrial processes as well as land use change.

## References:


[1] "H2A: Hydrogen Analysis Production Models." National Renewable Energy Laboratory (NREL), version 3.2018, 2018. https://www.nrel.gov/hydrogen/h2a-production-models.html.

[2] Muratori, Matteo, Catherine Ledna, Haewon McJeon, Page Kyle, Pralit Patel, Son H. Kim, Marshall Wise, Haroon S. Kheshgi, Leon E. Clarke, and Jae Edmonds. "Cost of Power or Power of Cost: A U.S. Modeling Perspective." Renewable and Sustainable Energy Reviews 77 (September 1, 2017): 861–74. https://doi.org/10.1016/j.rser.2017.04.055.

[3] Schmidt, O., A. Gambhir, I. Staffell, A. Hawkes, J. Nelson, and S. Few. "Future Cost and Performance of Water Electrolysis: An Expert Elicitation Study." International Journal of Hydrogen Energy 42, no. 52 (December 28, 2017): 30470–92. https://doi.org/10.1016/j.ijhydene.2017.10.045.

[4] Vimmerstedt, Laura, Sertac Akar, Chad Augustine, Philipp Beiter, Wesley Cole, David Feldman, Parthiv Kurup, et al. "2019 Annual Technology Baseline ATB Cost and Performance Data for Electricity Generation Technologies." National Renewable Energy Lab. (NREL), Golden, CO (United States); Oak Ridge National Lab. (ORNL), Oak Ridge, TN (United States), July 24, 2019. https://doi.org/10.11578/1544562.

[5] "H2A: Hydrogen Analysis Production Models." National Renewable Energy Laboratory (NREL), version v2.1.1, 2008. https://www.nrel.gov/hydrogen/h2a-production-archive.html.

[6] Timmerberg, Sebastian, and Martin Kaltschmitt. "Hydrogen from Renewables: Supply from North Africa to Central Europe as Blend in Existing Pipelines – Potentials and Costs." Applied Energy 237 (March 1, 2019): 795–809. https://doi.org/10.1016/j.apenergy.2019.01.030.

[7] "Net Zero by 2050." International Energy Agency (IEA), 2021. https://www.iea.org/reports/net-zero-by-2050.

[8] "The Future of Hydrogen." International Energy Agency (IEA), 2019. https://iea.blob.core.windows.net/assets/9e3a3493-b9a6-4b7d-b499-7ca48e357561/The_Future_of_Hydrogen.pdf.

[9] "Global Hydrogen Trade to Meet the 1.5°C Climate Goal: Part II – Technology Review of Hydrogen Carriers." International Renewable Energy Agency (IRENA), 2022. https://www.irena.org/publications/2022/Apr/Global-hydrogen-trade-Part-II.

[10] Quarton, Christopher J., and Sheila Samsatli. "Should We Inject Hydrogen into Gas Grids? Practicalities and Whole-System Value Chain Optimisation." Applied Energy 275 (October 1, 2020): 115172. https://doi.org/10.1016/j.apenergy.2020.115172.

[11] "Hydrogen Delivery Scenario Analysis Model (HDSAM)." Argonne National Laboratory. version 3.1 https://hdsam.es.anl.gov/index.php?content=hdsam.

[12] Bond-Lamberty, Ben, Pralit Patel, Joshua Lurz, Page Kyle, et al. "JGCRI/Gcam-Core: GCAM 6.0." Zenodo, June 7, 2022. https://doi.org/10.5281/zenodo.6619287.

[13] Byers, Edward, Volker Krey, Elmar Kriegler, Keywan Riahi, Roberto Schaeffer, Jarmo Kikstra, Robin Lamboll, et al. "AR6 Scenarios Database (v1.1)." Intergovernmental Panel on Climate Change, November 9, 2022. https://doi.org/10.5281/zenodo.7197970.

[14] McJeon, Haewon, Bryan K. Mignone, Patrick O'Rourke, Russell Horowitz, Haroon S. Kheshgi, Leon Clarke, Page Kyle, Pralit Patel, and Jae Edmonds. "Fossil Energy Deployment through Midcentury Consistent with 2°C Climate Stabilization." Energy and Climate Change 2 (December 1, 2021): 100034. https://doi.org/10.1016/j.egycc.2021.100034.

[15] Fuhrman, Jay, Andres Clarens, Katherine Calvin, Scott C. Doney, James A. Edmonds, Patrick O'Rourke, Pralit Patel, Shreekar Pradhan, William Shobe, and Haewon McJeon. "The Role of Direct Air Capture and Negative Emissions Technologies in the Shared Socioeconomic Pathways towards +1.5 °C and +2 °C Futures." Environmental Research Letters 16, no. 11 (October 2021): 114012. https://doi.org/10.1088/1748-9326/ac2db0.
